# Ultrathin, soft, radiative cooling interfaces for advanced thermal management in skin electronics

**DOI:** 10.1126/sciadv.adg1837

**Published:** 2023-04-07

**Authors:** Jiyu Li, Yang Fu, Jingkun Zhou, Kuanming Yao, Xue Ma, Shouwei Gao, Zuankai Wang, Jian-Guo Dai, Dangyuan Lei, Xinge Yu

**Affiliations:** ^1^ Department of Biomedical Engineering, City University of Hong Kong, Hong Kong, China.; ^2^ Hong Kong Center for Cerebra-Cardiovascular Health Engineering, Hong Kong Science Park, New Territories 999077, Hong Kong, China.; ^3^ Department of Materials Science and Engineering, The Hong Kong Institute of Clean Energy, City University of Hong Kong, 83 Tat Chee Avenue, Hong Kong, China.; ^4^ Department of Mechanical Engineering, City University of Hong Kong, Kowloon, Hong Kong, China.; ^5^ Department of Civil and Environmental Engineering, The Hong Kong Polytechnic University, Hong Kong, China.

## Abstract

Thermal management plays a notable role in electronics, especially for the emerging wearable and skin electronics, as the level of integration, multifunction, and miniaturization of such electronics is determined by thermal management. Here, we report a generic thermal management strategy by using an ultrathin, soft, radiative-cooling interface (USRI), which allows cooling down the temperature in skin electronics through both radiative and nonradiative heat transfer, achieving temperature reduction greater than 56°C. The light and intrinsically flexible nature of the USRI enables its use as a conformable sealing layer and hence can be readily integrated with skin electronics. Demonstrations include passive cooling down of Joule heat for flexible circuits, improving working efficiency for epidermal electronics, and stabling performance outputs for skin-interfaced wireless photoplethysmography sensors. These results offer an alternative pathway toward achieving effective thermal management in advanced skin-interfaced electronics for multifunctionally and wirelessly operated health care monitoring.

## Teaser

A generic thermal management strategy using a radiative-cooling interface can cool down the temperature in skin electronics.

## INTRODUCTION

The next generation of wearable devices could evolve into the format of skin-like electronics for direct integration with human skin (*1*–*4*). With their stable electrical response and outstanding mechanical tolerance, skin-integrated electronics has enabled various on-body applications, including biosignal monitoring (*5*–*7*), clinical treatment (*8*–*10*), human-machine interface (*11*–*13*), virtual reality, and augmented reality (*3*, *14*, *15*). Recent advances in materials development, device miniaturization, and system integration enable the performance of soft electronics to approach that of the traditional rigid electronics. Accordingly, effective thermal dissipation becomes a central topic in the emerging wearable and skin electronics (*16*, *17*). In electronic devices, heat could be either generated from internal electronic components or acquired from external sources such as light and hot air. Long-duration operation of electronic devices at high temperatures could lead to the deterioration of their electrical performance (*18*–*20*), shortened lifetime, and even the risk of skin burning (*21*, *22*), especially for those electronics with high power consumption and intense component integrations (*23*). Therefore, the investigation of heat dissipation in skin electronics becomes urgent, as it would be the key to further improving their performance and integration capacity as well as opening up new applications.

To mitigate the immoderate heat in wearable electronics, conduction and convection are the traditional solutions that transfer the generated heat to the surrounding air or cooling liquids and thermoelectric materials (*24*–*27*). However, those additional cooling systems always require excessive volume and power consumption, which impair the portability and wearable property of the devices. Using materials with a high thermal conductivity as cooling components is another strategy, but, unfortunately, the intrinsic relationship between thermal conductivity and mechanical flexibility of general materials is the biggest hurdle, i.e., *k* ≈ (*E*/ρ)1/2(*C_V_ℓ*/3) (*28*, *29*), where *k*, *C_V_*, *E*, ρ, and ℓ represent thermal conductivity, volumetric heat capacity, elastic modulus, density, and the average mean free path of phonons, respectively (*30*). Therefore, integrating thermal interfacial materials (TIMs) with electronics could be a worth-considering solution, due to their features of large thermal conductivity, low electrical resistance, and high thermal-dissipation efficiency in a passive way. To date, various types of TIMs have been reported, including metallic materials [copper (*31*), aluminum (*25*), steel (*32*), and liquid metal-encapsulated elastomer (*29*, *33*)], nanomaterials [graphene (*34*, *35*), carbon nanotube (*36*, *37*), and boron nitride (*38*–*40*)], flexible phase-changing materials (*41*), and polymer-based composites (*42*). However, to some extent, they all suffer from specific limitations, for example, the bulky and rigid nature of metallic materials, the risk of liquid leakage for deformable liquid metals (*29*), the increased thermal resistance of nanomaterials and polymers due to stretching-releasing-induced deficient contact (*30*), and the limitations in mass production and thermal cyclability of flexible phase-changing materials (*41*).

The recent advances of radiative cooling materials enable dissipating thermal energy through electromagnetic radiation and have been widely applied in infrastructures, vehicles, etc. (*43*–*46*). Encouragingly, recent works have reported the feasibility of merging the radiative cooling technology with wearable electronics operating under sunlight (*16*, *47*), offering a new paradigm for thermal management in wearable or skin electronics. However, the infrared emissivity of the radiative cooling materials developed for wearable electronics was lower than 0.86, hindering the devices from unleashing the full potential of radiative cooling (*48*). Moreover, the nonradiative contribution on temperature cooling has been ignored, which is an equally important key issue for thermal management (*49*, *50*). It would be a revolutionary solution of thermal management in skin electronics if one can design a radiative cooling material with both ideal radiative property and adequate nonradiative heat transfer and integrate it with soft electronics in an easy-to-implement manner.

Here, we report a generic thermal management strategy for soft electronics by integrating an ultrathin, soft, radiative cooling interface (USRI) with skin electronics for both radiative heat transfer and nonradiative heat dissipation. The USRI is a micrometer-thick polymeric coating layer that exhibits near-unit infrared emittance and high solar reflectance, as well as robust mechanical flexibility. Skin-like electronic devices coated with the USRI show significant improvement in thermal management, with the maximum temperature reduction of 56°C observed during operation. The intrinsically flexible nature of the USRI allows the electronics to undergo stable cooling even under extreme deformations including bending, twisting, folding, and stretching. With the efficient passive cooling capacity and the sophisticated nonradiative thermal design, the performance of the skin electronics, including the efficiency of wireless power transfer to light emitting diodes (LEDs) and the sensing signal stability under environmental obstructions (sunlight, hot wind and water), is significantly improved.

**Fig. 1.**
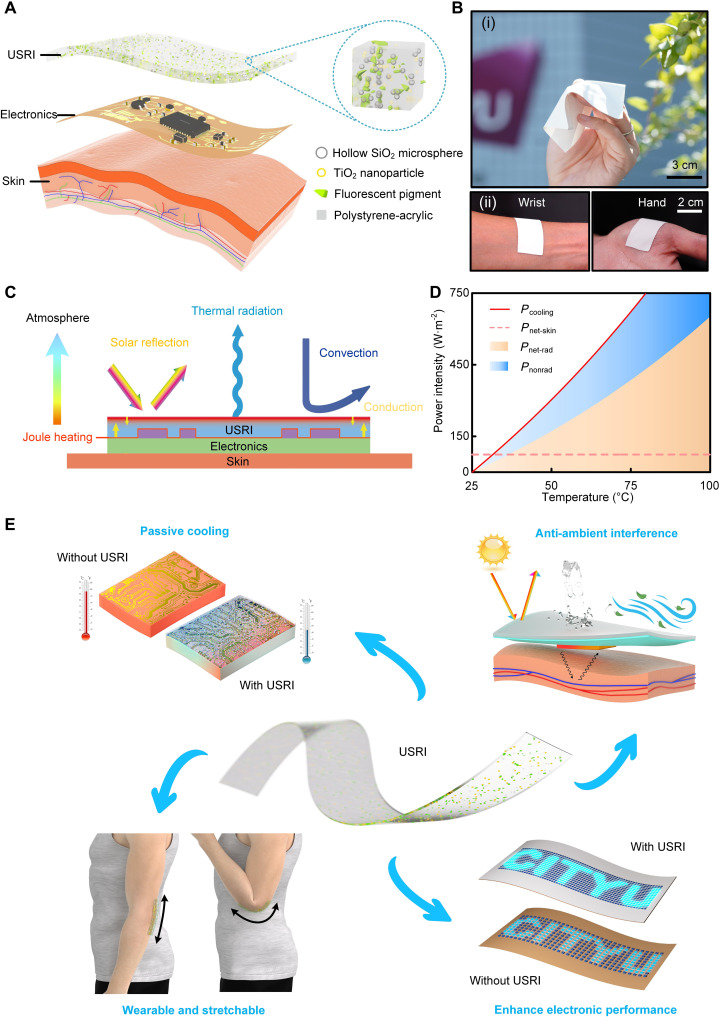
Overview of the USRI-enabled thermal management for wearable electronics. (**A**) Explosive view of the components and assembly method of the USRI. (**B**) Photographs of a fabricated USRI layer (i) and that attached on the wrist and hand (ii). (**C**) Thermal exchange processes in wearable electronics seamlessly integrated with a USRI, including radiative (thermal radiation and solar reflectance) and nonradiative (convection and conduction) contributions, as well as the internal Joule heating. (**D**) Comparison of cooling power from the radiative and nonradiative processes in wearable devices as a function of the above-ambient temperature caused by Joule heating. (**E**) Conceptual graph capturing functional advantages and potential applications of USRI in wearable and stretchable electronics.

Figure 1A shows the structure and composition of our USRI developed for achieving effective thermal management in wearable devices, which consists of hollow SiO_2_ microspheres for improving the infrared radiation, rutile TiO_2_ nanoparticles for enhancing the solar reflection, and fluorescent pigments for converting the absorbed ultraviolet (UV) light into visible light. After optimizing the proportions of main constituents (see note S1 for details), our USRI reveals high infrared emissivity, good mechanical property, and low thermal conductivity, which suits soft electronics better than previously reported cooling coating with the same material system (*51*). The lightweight (1.27 g/cm^2^) and flexible features of the USRI allow direct interfacing with skin for most parts of the human body, without causing any allergic response (Fig. 1B and fig. S5, A and B). Furthermore, the integration of USRI with skin electronics is highly compatible with existing microfabrication processes such as spin coating, laser cutting, and mask-spray patterning (fig. S6, A to C). By simply coating the USRI onto wearable devices as a conformable sealing layer, we observe significant temperature reduction and performance improvement of the devices under realistic operating conditions, which are mainly attributed to the radiative thermal dissipation. Figure 1C illustrates the thermal exchange processes occurring in a USRI-coated wearable device, where both the radiative (thermal radiation, solar irradiation) and nonradiative (convection, conduction) heat transfer processes contribute to the thermal dissipation of the coated electronics. The USRI enables strong thermal radiation and high solar reflectance to achieve an upward net heat flow (i.e., cooling effect) applicable for both indoor and outdoor applications. According to the Newton-Stefan cooling model, the radiative heat transfer becomes more significant as the device temperature rises since it obeys the *T*^4^ law [rather than the linear relationship governing the nonradiative heat transfer; (*52*)]. As shown in Fig. 1D, the cooling power intensity generated from radiative heat transfer becomes larger than 75 W/m^2^ as the device temperature surpasses the skin temperature, which is superior to nonradiative methods for passive heat dissipation. In view of this observation, a collection of device demonstrations is presented in this work to demonstrate the extraordinary thermal management capacity of USRI-integrated wearable electronics, as conceptually illustrated in Fig. 1E. First, applying a thin USRI layer of a few hundreds of micrometer thickness can significantly reduce the temperature of soft electronics by effectively dissipating the internal heat source, especially under high power input. Furthermore, ascribing to the crucial cooling effect, the efficiency of power transport can be improved for wireless epidermal electronics, thereby increasing the electrical performance of the USRI-coated device. Besides, under external heat sources, enhanced signal stability and device temperature reduction can be obtained. Last, the wearability and stretchability of the USRI are fully revealed through all the device demonstrations. Overall, our USRI may pave the way to realize unprecedented thermal management in advanced wearable electronics such as integrated circuits, high-power consumption electronics, and multifunctional wireless epidermal electronics.

## RESULTS

### USRI design and characterization

**Fig. 2.**
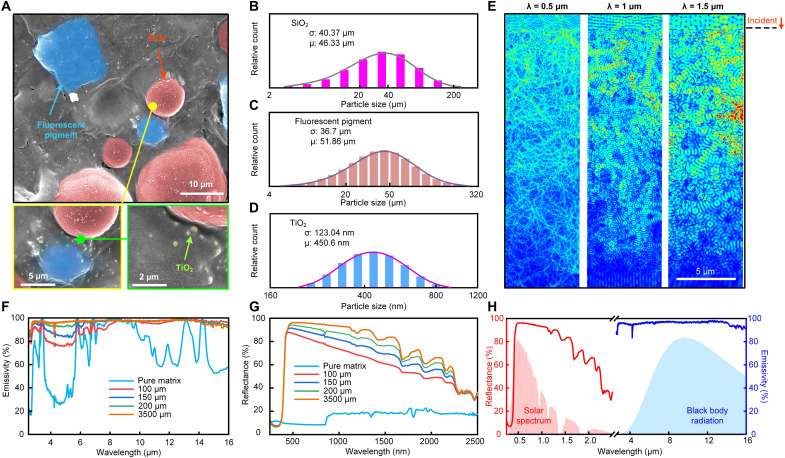
Structural and optical characterizations of the USRI. (**A**) Cross-sectional SEM micrograph of the USRI with all functional fillers homogeneously mixed in a polymer matrix. (**B** to **D**) Size distribution histograms of SiO_2_ microparticles (B), fluorescent pigments (C), and TiO_2_ nanoparticles (D). (**E**) Electromagnetic simulations of electric field distribution in the USRI under light illumination at wavelengths of 0.5, 1, and 1.5 μm. (**F** and **G**) Infrared emissivity (F) and solar reflectance (G) of USRIs with varied thickness, in comparison with that of a pure polymer matrix (pure matrix). (**H**) Solar reflectance and infrared emissivity spectra of a 3500-μm-thick USRI.

To facilitate wearable electronics–compatible thermal management technologies, a composite polymer with radiative cooling capacity was adopted for designing USRI because of its flexibility, multifunctionality, and cost-effectiveness (*53*, *54*). The cross-sectional view of the designed USRI is shown in Fig. 2A, which consists of a polymer matrix (poly-styrene-acrylic) and three functional fillers (hollow SiO_2_ microspheres, TiO_2_ nanoparticles, and fluorescent pigments). It can be observed that all functional fillers were randomly dispersed in the polymer matrix. The polymer matrix provides preliminary mid-to-far infrared emission through vibrational and rotational modes of molecular bonds including C─O─C, C═C, C─O, and C═O, which enable absorption peaks either in or out the atmospheric window (8 to 13 μm). Both rutile TiO_2_ nanoparticles and hollow SiO_2_ microspheres were applied to boost the infrared radiation from intrinsic phonon modes, while the inner air core of hollow SiO_2_ microspheres reduces the weight of the interfaces to improve the wearability and also decrease the thermal conductivity of the whole layer to combat the obstruction of external heat sources (see table S1 and fig. S7, A to D). Besides infrared contribution, TiO_2_ nanoparticles were applied to significantly improve the solar reflectance of USRI due to multiple Mie scattering (*55*). To compensate for the intrinsic UV absorption of TiO_2_, fluorescent pigments (SrAl_2_O_4_:Eu^2+^, Dy^3+^, and Yb^3+^) were introduced to compete with TiO_2_ on UV absorption and convert the absorbed UV light to re-emitted visible light (*56*, *57*). TiO_2_ nanoparticles at the diameter around 200 to 500 nm perform the highest scattering efficiency for incident light with wavelength at around 450 to 750 nm, matching the highest region of solar intensity (*58*). The large difference between the dimensions of TiO_2_ nanoparticles and fluorescent pigments allows more efficient UV absorption reduction and effective solar reflectance improvement (*57*). Whole components in the USRI with negligible degradation exhibit a high chemical stability and strong durability after exposure based on previous studies (*56*, *59*, *60*). Therefore, a Gauss-like size distribution (Fig. 2D) with a center diameter of ~450 nm was selected to achieve efficient multiple scattering of the sunlight. The size distributions of other functional fillers are also shown in Fig. 2 (B and C). The electric field distribution in Fig. 2E reveals the propagation of incident light for different wavelengths. It can be observed that incident light within the solar spectrum is obviously weakened after penetrating the interfaces for several tens of micrometers, leading to efficient backscattering. Figure 2 (F to H) reveals the spectral properties of USRI at different thicknesses (100 to 3500 μm) in comparison with pure polymer matrix. It can be observed that the polymer matrix is emissive in infrared and highly transparent for the sunlight. After combining the functional fillers, the infrared emissivity and solar reflectance were both significantly boosted (table S2). Since the device temperature is always higher than the ambient due to the internal heat sources (mainly Joule heat from the circuit), our USRI performs a broadband infrared emission to increase outgoing infrared radiation rather than a selective emission for subambient radiative cooling (*61*). Moreover, a thicker USRI exhibits better spectral properties as it provides more scattering interfaces as well as more emissive substances. The overall infrared emittance of 200-μm-thick USRI is 97%, which is superior to the previously reported radiative cooling materials and conventional encapsulation layers [e.g., polyimide (PI)]. For example, the infrared emittance of a 50-μm-thick USRI surpasses that of a 500-μm-thick PI (commonly used in wearable electronics; fig. S3). Considering the thermal stability requirement under external environmental obstructions, hollow SiO_2_ microspheres should be well encapsulated within the cooling interface. Therefore, the thickness limit of the USRI should be >40 μm, referring to the size distribution of hollow SiO_2_ microspheres shown in Fig. 2B. Note that only solar reflectance spectra without the fluorescent contribution are shown here since commercial UV-visible near-infrared (UV/VIS/NIR) spectrometers cannot distinguish the reflected light at incident wavelength from fluorescence emission at another wavelength (*62*). The effective solar reflectance of a 3500-μm-thick USRI reaches more than 91% by considering the fluorescent contribution (*62*) (see Methods and fig. S8).

### Passive cooling of conductive interconnects (generating Joule heat) in skin electronics

**Fig. 3.**
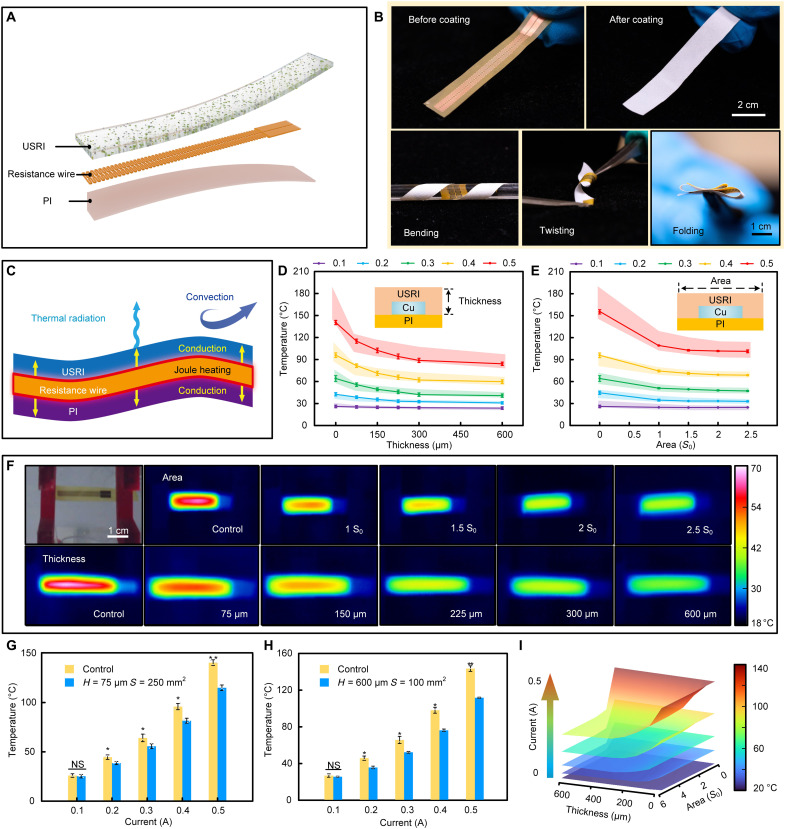
Passive cooling for conductive interconnects in skin electronics. (**A**) Explosive view of a USRI-integrated flexible heating wire. (**B**) Photographs of the flexible heating wire before and after coating with the USRI, showing their seamless and robust integration under bending, twisting, and folding. (**C**) Thermal exchange processes of the USRI-coated flexible heating wire. (**D** and **E**) Measured temperature variation of the USRI-integrated flexible heating with varied interface thickness (D) and interface area (E) under different working currents. The colored shaded regions depict simulation results. (**F**) Image of the USRI-integrated flexible heating wire and corresponding infrared images of such devices with different thicknesses and areas. The working current was kept at 0.3 A. (**G** and **H**) Statistics of cooling temperatures of two USRI-coated flexible heating wires working at a current varying from 0.1 to 0.5 A. Both the thickness and the interface area present significant differences between the control and USRI groups (*P* = 0.012847 for interface thickness, *P* = 0.020245 for interface area, *n* = 3). (**I**) Temperature distribution of USRI-integrated flexible heating wires with varied thickness, area, and current.

**Fig. 4.**
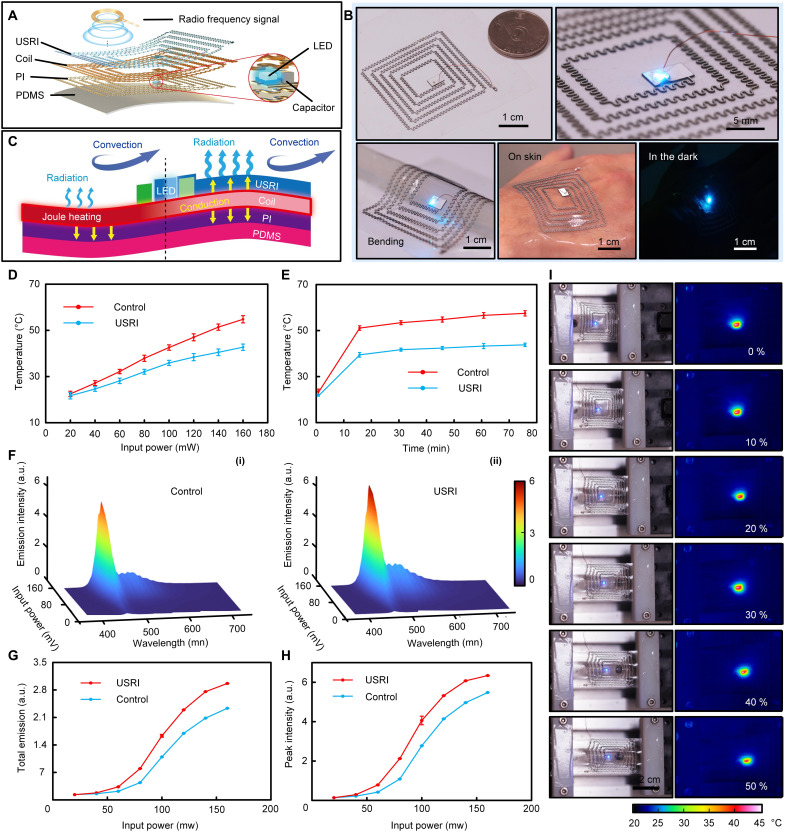
Passive cooling for stretchable RF wireless-based skin electronics. (**A**) Explosive view of a USRI-integrated wireless stretchable epidermal lighting system. (**B**) Photographs of the USRI-integrated epidermal lighting system working under bending, on skin, and in the dark. (**C**) Thermal exchange processes in the USRI-integrated epidermal lighting system. (**D**) Temperature variations in the USRI-integrated epidermal lighting system and a control device with increasing input power. The USRI has a thickness of 225 μm. (**E**) Temperature evolution in the USRI-coated epidermal lighting system under long-term stretching (5 to 50%) for 75 min. (**F**) Emission distribution of the fluorescent light generated from the epidermal lighting systems: control group (i) and USRI group (ii). (**G** and **H**) Total emission intensity (G) and peak intensity (H) of the fluorescent light generated from the two devices in (F). (**I**) Optical and infrared images of the USRI-coated epidermal lighting system working under stretching from 0 to 50%.

The radiative cooling capacity of the USRI on dissipating Joule heat produced by conductive interconnects in skin electronics was first investigated, where metallic traces with typical structural design served as resistance wires since the Joule heat from resistance wires contribute to most temperature increase in electronics. The schematic diagram of the soft device is shown in Fig. 3A, where the resistance wires (area: *S*_0_ = 100 mm^2^; fig. S9A) for Joule heat generation is sandwiched between the polymer substrate (PI) and the top USRI layer. The USRI-integrated flexible resistance wires exhibit great flexibility due to the intrinsically soft nature of the USRI and well-designed mechanical structures of the metallic traces. Moreover, plasmonic treatment improves the van der Waals bonding strength and thereby the adhesion between the flexible resistance wires and the USRI under bending, twisting, and folding (Figs. 3B and 4B and table S3). As shown in Fig. 3C, the Joule heat generated from the wires flows to the USRI layer and dissipates to the ambient environment through air convection and thermal radiation. Although thermal conduction also occurs toward the polymer substrate and the underlying skin, the open space above the USRI provides a cooler radiative heat sink and an additional thermal exchange channel (i.e., convection), which is more efficient and favorable for heat dissipation. Therefore, the adoption of the USRI can significantly reduce the heat flow toward the underlying skin, thus improving thermal comfortability and reducing the risk of skin burns. A control group of devices with bare resistance wires on the PI substrate was also prepared and tested as a comparison (Fig. 3, D to I), where the convection remained, while thermal radiation was significantly reduced because of the low emissivity of metals. To evaluate the cooling effect of the USRI, we measured the temperature variations of the flexible resistance wires for both the control group and the group coated with USRI under a series of coating thickness (*H* = 75 to 600 μm at fixed coating area: *S* = 1.5 *S*_0_) and coating areas (*S* = 1 to 2.5 *S*_0_ at fixed coating thickness: *H* = 150 μm). The temperature of the PI substrate was measured, which is the accurate temperature of the device, since there may be some unexpected influence of variables such as the emissivity difference induced by the interface’s thickness variation. It is well known that the heat generated by electronics is associated with the input power. Here, the influence of input current ranging from 0.1 to 0.5 A to the flexible resistance wires on the temperature change was studied, where the data were collected 5 min after reaching the thermal equilibrium state (fig. S9B). As shown in Fig. 3 (D and E), when the flexible resistance wires work at lower input current (0.1 A), there is no obvious temperature difference between the USRI-coated group and the control group (i.e., *H* = 0) due to the ignorable Joule heat generation. Along with increasing the input current, the temperature variation is significant, where the USRI-coated group shows a much lower temperature (Fig. 3, D and E). These results are highly consistent with computational simulation results (colored shaded region; Fig. 3, D and E). For those devices with fixed coating areas, thicker USRI layers render the improvement of overall emissivity for a better cooling effect, while for a specific coating thickness, the heat of the conductive resistance wires wrapped in the USRI spreads horizontally. The infrared images reveal that increasing both coating area and thickness can lead to a better cooling effect, the hot region slightly expanded with increased coating area (Fig. 3F), and the cooling effect remains the same even under deformation (fig. S9C). The low thermal conductance of the USRI may limit the expansion of the highly emissive area and constrain the cooling effect (fig. S10B); however, it will reduce the resistance variation at high input power (fig. S11) and improve device performance in the presence of external heat source. Figure 3 (G and H) shows further comparisons between the USRI group and the control group. As the coating thickness is fixed at *H* = 75 μm, the temperatures of the USRI group with a coating area of *S* = 2.5 *S*_0_ (32.9°, 47.2°, 68.73°, and 101.3°C) are obviously lower than those of the control group (44.6°, 64.1°, 95.9°, and 140.5°C) with the current ranging from 0.2 to 0.5 A, while as the coating area is fixed at *S* = *S*_0_, the temperatures of the USRI group can be further lowered down to 0.2 A at 30.9°C, 0.3 A at 40.8°C, 0.4 A at 59.8°C, and 0.5 A at 84.2°C with *H* = 600 μm. Moreover, the USRI outperforms common encapsulation layers (e.g., PI) in cooling capacity due to its higher infrared emissivity. It is worth mentioning that the USRI also exhibits robust performance stability over a 600-min continuous test of 100 heating/cooling cycles (fig. S12B). Since thermal comfortability is one of the most critical issues for skin-interfaced electronics, it is necessary to constrain the device temperature below 44°C to avoid thermal discomfort or skin burns. As shown in Fig. 3I, the flexible resistance wires with USRI can cool down the temperature from 64.1°C (the control group) to 42.12°C at the input current of 0.3 A with the coating thickness of 150 μm. Therefore, the USRI provides an excellent thermal management strategy for wearable electronics.

### Passive cooling for stretchable RF wireless-based skin electronics

Stable thermal management for skin electronics under deformation is essential in various applications, especially in long-term biosignal monitoring and wireless communication. Wireless technologies, especially radio frequency (RF)–based techniques, enable battery-free skin electronics and get rid of many external connections (*5*, *17*, *63*). It is well known that long-term working under RF leads to significant thermal concerns in electronics, which is also the hurdle for RF-based wireless skin electronics. Here, we developed wireless stretchable skin electronics with a lighting system to further demonstrate the thermal management capacity of the USRI for potential applications. This wireless electronic device consists of a serpentine coil as RF antenna for wireless power transmission, an LED (with capacitor), a PI-supporting layer, and a bottom polydimethylsiloxane (PDMS) as sealing layer and substrate (Fig. 4A). The patterned USRI (with a thickness of *H* = 225 μm after considering the cooling capacity and aspect ratio of the serpentine pattern integrated with USRI) serves as both an encapsulation layer and a thermal management cover on top of the device. Figure 4B shows the optical images of the USRI-coated wireless electronic device, where the device exhibits great flexibility that allows providing stable working status even under bending, stretching, or even integrating with skin (fig. S13). Figure 4C shows the schematic illustration of the thermal exchange processes for this flexible wireless electronic device. Similar to the thermal management behaviors in the flexible resistance wires, here, the USRI also provides a heat dissipation channel much superior to the devices without USRI, enabling efficient cooling of the USRI-integrated system. To evaluate the thermal management capacity of the USRI on wireless electronics, temperature variation of the PDMS substrate was recorded first for the devices continuously working at the input power ranging from 20 to 160 mW for 15 min. As shown in Fig. 4D and fig. S14, the temperature of the USRI group is much lower than that of the control group at all input powers. The cooling effect is amplified by the increase of the input power, which is consistent with the results for flexible resistance wires. Figure 4E reveals the temperature variation as a function of time for both the USRI and control groups (input power of 160 mW). It can be seen that the temperature of the USRI group approaches thermal equilibrium within 20 min, while the temperature of the control group rose slightly after that. Therefore, the temperatures in Fig. 4D can be approximately regarded as equilibrium states.

The radiative cooling effect enabled thermal management in the USRI-coated electronics, which can benefit the working performance of the skin electronics. Here, the spectral emission intensities of the LEDs in the devices serving as the index are used to evaluate both groups of devices. Figure 4F shows the emission intensities difference of the LEDs in both groups at different input powers ranging from 20 to 160 mW. Significant improvement of illumination intensity with restrained envelope can be observed for the USRI group. Further comparisons of total emission and peak intensity for the two groups are shown in Fig. 4 (G and H). The total emission (integrated from 350 to 720 nm) and peak intensity (at 462.7 nm) of the USRI group show a noticeable improvement of 15.6 and 27.3%, respectively, compared to the control group at the input power of 160 mW, while the improvement of the total emission and the peak emission intensity of the LED with PI as the encapsulation layer is 7.1 and 9.8%, respectively (fig. S15), which is much lower than that of the USRI encapsulated device, indicating the superiority of the USRI to improve the emission efficiency. Therefore, the USRI renders a higher illumination intensity of the LED and a higher energy conversion efficiency for this wireless lighting system. The reason is straightforward: The decreased device temperature reduces the resistance of the circuit and thereby releasing more energy from Joule heating to lighting. Therefore, our USRI not only can provide considerable cooling effect but also improved power efficiency for skin electronics.

It is worth mentioning that the USRI-coated devices not only exhibit good thermal management capacity but also maintain excellent stretchability. Evidence can be found in fig. S16. The temperature measurement for the wireless electronic device under a stretching range from 5 to 50% for 1000 times (input power of 160 mW) is revealed in Fig. 4I, exhibiting stable performance during stretching. Note that the thermal equilibrium temperature of 43.7°C for the USRI group without stretching is much lower than that of the control group (56.7°C) (Fig. 4E). While the device works under stretching state at 50% (Fig. 4I), the temperature of the USRI group (~42.7°C) remains close to 43.7°C at the same input power. The small temperature reduction of stretched devices may originate from the enlarged area under deformation. Both illumination intensity and device temperature of the USRI-integrated lighting system exhibited no apparent variation under different stretching, indicating a stable thermal management strategy.

### Passive cooling for continuous monitoring of physiological signals in skin electronics

**Fig. 5.**
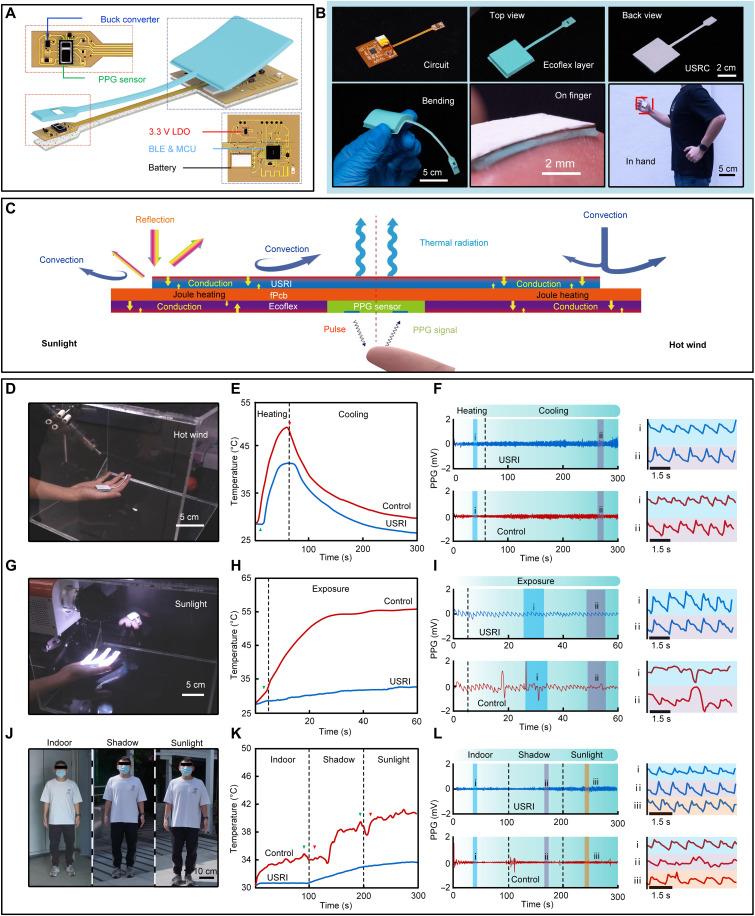
Passive cooling enhanced continuous physiological signal monitoring in skin electronics. (**A**) Explosive view of a USRI-coated finger-like PPG sensing system. (**B**) Top view and back view of the fabricated device and photographs of the device under bending and attaching on a fingertip. (**C**) Thermal exchange processes in the device. (**D** to **L**) Photographs of the device working under hot wind (D) and sunlight exposure (G) as well as moving from indoor to shadow and sunlight (J), and corresponding temperature variations (E, H, and K) and PPG signals (F, I, and L) monitored from the fingertip.

In addition to the internal heat source (i.e., Joule heat) of the electronics, the external heat sources (i.e., ambient sunlight and hot wind) should not be overlooked, as they may lead to severe electrical performance degradation and restrict the outdoor applications of skin electronics. To fully exploit the practical applications of skin electronics by our thermal management strategies, we developed a state-of-the-art skin-interfaced photoplethysmography (PPG) wireless sensing platform for real-time pulse monitoring and evaluating the influence of the USRI on the device performance. As shown in Fig. 5 (A and B), the skin-interfaced PPG sensor consists of a PPG sensor (MAX30102, typical working current: 10 to 40 mA), a microcontroller unit (MCU, CC2640R2F), a battery-based power supply, a bluetooth wireless module, and other electronic components. The top surface of the PPG sensor was coated with a 0.5-mm-thick USRI, and the device can be applied to the fingertips for PPG wireless monitoring. In an outdoor or harsh environment, both sunlight and hot wind can heat up the device very fast. Therefore, solar absorption and thermal conduction should be constrained to prevent heat generation besides boosting thermal radiation for heat dissipation. As shown in Fig. 5C, the low thermal conductive feature of the USRI can slow down thermal exchange between external heat source and the device, preventing the system from rapid temperature variations and thus improving the performance stability of the system. Meanwhile, it is convenient to modify the interface surface with hydrophobic property to achieve effective water/sweat resistance features, which for sure would improve the stability and functionality of wearable devices (fig. S17). To investigate the potential of the USRI for combatting the obstruction caused by external heat sources, both PPG signal and device temperature were recorded under two situations: exposed under a sun simulator (Fig. 5, D to F) and blown by hot air (Fig. 5, G to I). The power intensity of the sun simulator is 1000 W/m^2^. The air temperature and flow rate of the wind were controlled at 55°C and 5 liters/min, respectively. As shown in Fig. 4 (E and H), the peak values of the device temperature with USRI are 40.4° and 31.8°C under hot wind and sun exposure, respectively, which are much lower than the temperatures of the control group (hot wind: 48.9°C, sun exposure: 58.7°C). For the situation of exposure to sunlight, the USRI-coated device can reflect sunlight efficiently to reduce the solar heating and suppress the device temperature. As hot wind presents, the hollow glass microparticles in the interfaces act as thermal barriers that can weaken thermal conduction to prevent heat transfer from the external source heated top surface to the inner device. Therefore, the USRI can decrease the device temperature that improves the stability and accuracy of PPG signals as the light sources for signal acquisition are susceptible to the device temperature (fig. S18). Figure 5 (F and I) reveals the PPG signal recorded under external heat sources. It can be observed that the signal fluctuation of the USRI group is smaller than that of the control group. After further magnifying the signal (fig. S19), three distinct peaks are still very clear for the USRI group, while these peaks vanish for the control group. The missing characteristic peaks and aperiodic change of the PPG signals in the control group indicate that the control group fails to monitor PPG signals in the outdoor ambient environment. Last, the PPG sensing platform was applied to monitor the heart rate of a volunteer, who walked from indoor to outdoor (Fig. 5J). As shown in Fig. 5K and fig. S20, the temperature of the USRI group (33.8°C) is significantly lower than that of the control group (42.1°C) outdoors. Meanwhile, the plot of temperature for the control group is drastically fluctuated because of the wind, resulting in PPG signal distortion (Fig. 5L) and heart rate underestimation (table S3). In contrast, the PPG signal in the USRI group is more stable and distinct while walking, which indicates that the radiative thermal regulation together with nonradiative thermal design can efficiently reduce the obstruction from external environment. In the presence of the USRI, the wind directly heats up the outer surface of the USRI and consequentially inputs heat to the device through thermal conduction within the USRI layer. The low thermal conductivity of our USRI will impede the latter thermal process in the presence of external heat sources and thereby decrease the temperature fluctuation of the device.

## DISCUSSION

In conclusion, the results shown in this work highlight the materials, devices, and integration strategies of the USRIs for thermal management in skin electronics. To fully address the thermal management capacity, the USRI presents a near-ideal broadband infrared emissivity as well as an excellent solar reflection. We systematically investigated the cooling effect, wearability/stretchability, and performance improvement for a collection of wearable devices for the applications including cooling resistance wires, improving device performance, integration with wireless communication, and continuous stable physiological signal monitoring. The results reveal that significant temperature reduction can be obtained with a very simple cooling interface to achieve an excellent radiative cooling effect. Furthermore, USRI is also compatible with the well-established mechanical design in skin electronics, as it not only can improve the device performance and energy efficiency for wireless skin electronics but also maintains the excellent flexibility and stretchability of the state-of-the-art skin electronics. The lowered thermal conductivity of the cooling interfaces enables superior anti-interference ability upon environmental obstructions (i.e., external heat sources) that can sufficiently suppress signal fluctuation without scarifying too much on cooling effect. All of these demonstrations show great potential of the cooling interfaces in skin electronics and wearable technologies. Thus, the concept of using self-cooling interfaces provides a remarkable thermal management strategy for further advancement of highly integrated, multifunctional, and wireless wearable electronics in medical, communication, and entertainment applications.

## METHODS

### Preparation of USRI emulsion

The USRI emulsion was prepared through the mixture of polymer matrix emulsion and functional fillers (volume fractions of main components shown in table S5). First, 90 g of waterborne poly-styrene-acrylic emulsion (EC702, BASF Co. Ltd.) was added into the beaker, followed by ~20 g of grinding beads for mixing and stirring at a speed of 800 revolutions per minute (rpm) for 30 min. During the stirring process, functional fillers, including 40 g of TiO_2_ nanoparticles (Ti-PureR902, DuPont), 30 g of fluorescent pigments (4sx, Shenzhen YaoDeSheng Technology Co. Ltd.),and 6 g of hollow glass microparticles (K25, 3M), and 15 g of water were added in order. Four additives, including 5 g of PTFE powder (D50, Dongguan Zhanyang Polymer Co. Ltd.), 3 g of dispersant agent (SN5040, SAN NOPCO LIMITED), 2 g of antifoaming agent (NXZ, SAN NOPCO LIMITED), and 8 g of film-forming agent (TEXNOL, Eastman Chemical Company), were also added into the mixture to improve the performance quality of the interfaces. The mixture was then stirred at a speed of 400 rpm for 20 min. The final emulsion was obtained by filtering the grinding beads. All the interfaces used for experiments were spin-coated on the devices and baked on a hotplate at 70°C for 30 min.

### Characterization of USRI coating

The cross section of USRI coating was characterized by an FEI Quanta 450 FESEM after the USRI broke off when immersed into liquid nitrogen for 30 s. The size distributions of functional fillers were obtained using a Malvern Mastersizer 3000 Particle Size Analyzer. The spectral solar reflectance and infrared emissivity of USRI and pure polymer matrix were measured by a PerkinElmer Lambda 1050+ UV/VIS/NIR Wide Band Spectrometer (equipped with an integral sphere) and a Bruker Vertex-70 FITR spectrometer. The USRI was placed in a dark box for more than 8 hours to release the afterglow of fluorescent pigments before measuring the solar reflectance. The thickness of USRI was measured by a Bruker Dektak XT Profilometer. The mechanical properties including Young’s modulus, strain stress, toughness, and peel force were obtained using an Instron 5942 Micro Newton Tester. The strain-stress curve of a USRI layer (length: 50 mm, thickness: 200 μm, and width: 2 mm) was recorded under a stretching speed of 30 mm/min. The peel forces between the USRI layer (thickness: 150 μm) and a series of typical substrate/circuit materials (copper, Ecoflex, PDMS, PI, and flexible heating wire with a diameter of 5 × 50 mm) were measured by the 90° peel adhesion test at the speed of 20 mm/min.

### Processing routes for USRI-integrated flexible conductive interconnects

The schematic illustration of the fabrication process is shown in fig. S21. Fabrication began with spin-coating (3000 rpm, 30 s) a layer of photoresist (AZ 5214, AZ Electronic Materials) on a Cu (18 μm)/PI (30 μm) foil, followed by soft baking at 115°C for 5 min. After UV exposure for 10 s with a mask of the pattern, the photoresist was developed in AZ 400K solution for 90 s and subsequently baked at 115°C for 5 min. Afterward, the Cu layer was wet-etched by aqueous solution of FeCl_3_ for 2 min to remove the unwanted Cu, then rinsed with deionized (DI) water, and baked at 115°C for 5 min sequentially. Next, the pattern was rinsed using acetone for 1 min to remove unwanted photoresist. After soldering with enameled wire and plasma treatment (energy, 10 kJ; Harrick plasma cleaner PDC-002), the USRI was spin-coated onto the sample with deterministic rotational speed (the relationship between the thickness of USRI and rotational speed is shown in fig. S22) followed by baking at 70°C for 30 min. Last, another group of flexible resistance wire-coated with PI (thickness, 150 μm) was fabricated by multiple spin coating procedure (rpm: 500, baked at 250°C for 30 min, 15 times).

### Processing routes for USRI-integrated stretchable RF wireless-based skin electronics

The schematic illustration of the fabrication process is shown in fig. S23. Fabrication began with spin coating (600 rpm, 30 s) a layer of PDMS (PDMS:curing agent, 15:1) on a quartz glass slide (75 × 75 mm) followed by baking at 70°C for 5 min. Afterward, a foil of Cu (18 μm)/PI (30 μm) was paved on the layer of PDMS and then patterned by laser cutting (ProtoLaser U4; LPKF Laser & Electronics) to form a copper serpentine coil (35 × 35 mm, coil width: 180 μm; see fig. S24). Afterward, the LED (emission wavelength: 488 nm, typical working current: 25 to 60 mA) (*64*) and capacitor (70 pf) were soldered at the soldered dot. The enameled wire is thermally bonded with soldering paste at the connection port. Next, the top surface of the LED was tightly attached with tape (magic tape, 3M) as shielding mask. The area of shielding tape is laser-cut into the shape of the LED. After plasma treatment of foil, the USRI was sequentially spin-coated onto the coil at 200 rpm, and then the shielding tape is removed before baking at 70°C for 30 min as the USRI group. For the PI group, the coil was coated with PI (thickness: 225 μm) by multiple spin coating instead. Next, the fabricated coil integrated with USRI was patterned by laser cutting to remove the unwanted coating layer (the serpentine Cu coil is covered by the USRI only; see fig. S16C), so the major area uncovered by Cu/USRI is transparent. After laser cutting, the water-soluble tape (WST) was used to pick up the wireless stretchable E-skin lighting system. Depositing Ti/SiO_2_ on the bottom side of WST by E-beam forms the adhesive layer for strong bonding effect. Afterward, WST was treated by UV Ozone cleaner for 1 min together with another thin PDMS layer (PDMS:curing agent, 15:1; thickness: 75 μm) to form a chemical combination between the SiO_2_ layer and PDMS when the WST with the fabricated device mounted onto the PDMS. Last, the transferred pattern was immersed in water to remove the WST and then baked at 70°C for 30 min.

### Processing routes for USRI-integrated finger-like wireless PPG sensing platform

The schematic illustration of the fabrication process is shown in fig. S25. First, a customized flexible printed circuit board (fPCB; DingXin Ltd., China; fig. S26A) is prepared. The relative electrical components (i.e., PPG sensor, MCU, resistor, and low-dropout regulator) are soldered with solder paste on the circuit (fig. S26B). Then, the fPCB was put in the polyacrylate mold fabricated by three-dimensional (3D) printing using a commercial digital laser resin printer (HALOT-SKY, CRE-ALITY), and the surface of the mold was coated with a 5-μm layer of Parylene C by chemical vapor deposition using special Parylene deposition equipment (PDS 2010 421 Labcoter 2, Specialty Coating Systems Inc.) as an isolation molecular layer between mold and Ecoflex. Then, the uncured Ecoflex mixed with adding ~7 wt % blue dye (Silcpig) was poured into the mold (fig. S26C). The mold was covered by fPCB, the Ecoflex was cured at 60°C for 30 min in an oven, and then the Ecoflex-encapsulated fPCB was peeled off from the mold. After removing the redundant Ecoflex on the top side of fPCB and plasma treatment, the USRI was then spin-coated onto the top side of the fPCB with 200 rpm and baked at 70°C for 30 min. Before signal monitoring, the Ecoflex side of the device was attached seamlessly on the index fingertip of the volunteer using a liquid band aid to prevent sunlight interference.

### Mask-sprayed text pattern of USRI

The spraying mask began with laser cutting a 30-μm-thick layer of PI to pattern the text of “CITYU,” followed by rinsing in DI water for 5 min to remove the dust and then baking at 50°C for 10 min. Afterward, the spraying mask was attached seamlessly onto a PI film (Kapton; thickness: 0.13 mm) using tape. The USRI emulsion was then sprayed onto the mask for 20 min using a painter spray gun (LABEL, PR-01) and baked at 70°C for 30 min. After removing the spray mask carefully, the patterned USRI on the PI layer was rinsed in DI water for 5 min and then baked at 50°C for 10 min to remove the water.

### The wireless sensing platform design and operation

The design layer of this wireless sensing platform is shown in fig. S27; briefly, this wireless pulse oximeter and heart rate sensor consist of a PPG sensor (MAX30102, Maxim integrated, typical working current: 10 to 40 mA) (*65*), a bluetooth wireless MCU (CC2640R2F, TI), a low-dropout regulator (LDO) (TPS76933, TI), and a buck converter (TPS622314, TI; fig. S28). This module is powered by a lithium battery; the power from the battery will be converted to 3.3 V by the LDO to power the MCU and the LEDs in the PPG sensor (fig. S29). The buck converter will convert the 3.3-V voltage to 1.8 V as the analog power supply of the PPG sensor (fig. S30). The MCU can communicate with the PPG sensor through the interintegrated circuit bus and collect the data (fig. S31). After processing, the PPG signal information can be obtained. By using the Bluetooth Low Energy module built inside the MCU, the device can transmit the collected data to a mobile phone in real time.

### Temperature measurement method for flexible conductive interconnects, stretchable RF wireless-based skin electronics, and finger-like wireless PPG sensing platform

The temperature measurement method for flexible resistance wire and wireless stretchable epidermal light system is based on infrared measurement using a thermal imaging camera (FLK-TIS60, Fluke). Temperature variation of both devices was measured indoors. The temperature measurement was conducted in an enclosed environment to reduce interruption of thermal convection. The temperature measurement method for finger-like wireless PPG sensing platform was based on temperature sensing using the temperature sensor integrated inside the MCU. The measurement environment for the platform included indoor and outdoor situations.

#### Evaluation of cooling performance of USRI on flexible conductive interconnects

The experiment setup is shown in fig. S32. To investigate the cooling effect of USRI on the flexible heating wire, the fabricated flexible heating wire coating with USRI was attached using double-sided tape on a 3D-printed bracket. To reduce the thermal conduction between the heating wire and supporting bracket, the heating area of the device was suspended on the surface of the supporting bracket. The two poles of the heating wire were connected to a DC power supply using enameled wire. The temperature of the bottom side (PI layer) of the heating wire working at different currents (from 0.1 to 0.5 A) was measured by a thermal imaging camera. For the thermal stability test, the device temperature was recorded 3 min after turning on/off the power supply (input current: 0.3 A) over 100 heating/cooling cycles. The distance between the thermal imaging camera and the device was 30 cm.

#### Evaluation of the cooling effect of USRIon the stretchable RF wireless-based skin electronics

The experiment setup is shown in fig. S33. The wireless stretchable epidermal light system was tightly attached on a 3D-printed bracket. Also, to reduce the thermal conduction between the device and the supporting bracket, the working area of the fabricated device was directly in contact with air. The power supply for the epidermal lighting system was based on wireless RF technology. Briefly, the RF signal was generated by arbitrary waveform generators with the RF of 13.56 MHz based on the impedance analysis results of the lighting system (fig. S34); an RF coil (diameter: 35 × 35 mm, number of turns: 5) was applied as an RF antenna for power transmission. The settlement of RF coil was in the front of the lighting system at a center-to-center distance of 2 cm. The temperature in the backside (PDMS layer) was measured by a thermal imaging camera. The distance between the probe and the device was 30 cm.

For the temperature measurement of the epidermal lighting system under stretching, wired power supply was applied instead to reduce the influence of the diameter variation of the wireless coil on the power of the wireless RF method. The epidermal lighting system was directly connected to arbitrary waveform generators by enameled wire. Afterward, the epidermal lighting system was fixed (with the PDMS layer facing upward) at the instrument using a chuck at a device stretching range from 5 to 50% (speed: 7 mm/s). In fatigue stretching tests, the epidermal lighting system was stretched, ranging from 0 to 50% for 75 min (1000 times). The temperature on the PDMS layer was recorded by an RF probe after working for 15 min.

#### Evaluation of the cooling effect of USRIon the wireless finger-like PPG sensing platform

For the blown-by-hot-wind experiment, a hot air gun was applied to heat the back surface of the fabricated device during the signal monitoring (temperature of heat air: 55°C, flow rate: 5 liters/min). The experiment was executed for 300 s, including 60 s of heating and 240 s of cooling (Fig. 5, E and F) at the forearm. The distance between the air gun and the device is 30 cm.

For the temperature measurement of the device under sunlight exposure, a sunlight simulator (CEL-PF300-T10, CEAuLight Co. Ltd.) was applied to irradiate the back surface of the fabricated device with a power of 1000 W/m^2^ during signal monitoring. The power of the exposure was 1000W/m^2^, and the irradiation lasted for 60 s. The distance between the simulator and the device is 10 cm.

The temperature measurement for outdoor walking included three stages (indoor, outdoor under shade, and outdoor under sun exposure) during the monitoring of the PPG signal from the volunteer. First, the volunteer stands for 100 s indoors, then walks outside, and stands under a shade for 100 s. Then, the volunteer walks out of the shade and stands under the sun for 100 s. The hands of the volunteer drooped naturally during the monitoring of the PPG signal.

### Measurement of light intensities for the epidermal lighting system

The light intensities from the LEDs in the epidermal lighting system were measured through a customized upright microscope system (Olympus, BX51) (fig. S35). The LEDs for both the USRI group and the control group were fixed at the same height under the microscope. No objective was used since focused light would exceed the measurement range of the sensor. The light was directed by the microscope to a spectrometer (Princeton Instruments, SP2300i) coupled with a thermoelectic (TE)–cooled charge-coupled device (PIXIS: 400BR_eXcelon) for spectrum analysis.

### Calculation method for comparison between radiative and nonradiative heat transfer

The thermal exchange processes for wearable devices consist of radiative and nonradiative heat transfer, in which the cooling power intensity can be described as follows

$$P_{\mathrm{cooling}}=P_{\mathrm{net}-\mathrm{rad}}+P_{\mathrm{nonrad}}-P_{\mathrm{heat}}$$

Here, no evaporative thermal process is adopted since all the proposed devices are airtight. The net radiative and nonradiative cooling power intensity can be expressed as

$$P_{\mathrm{net}-\mathrm{rad}}=\epsilon (\sigma T^{4}-\epsilon_{\mathrm{amb}}\sigma T_{\mathrm{amb}}^{4})$$

$$P_{\mathrm{nonrad}}=h(T-T_{\mathrm{amb}})$$

where ϵ and ϵ_amb_ are the surface emissivity of the device and the effective emissivity of the ambient. σ and *h* denote the Stefan-Boltzmann constant and nonradiative heat transfer coefficient, respectively. *T* and *T*_amb_ are the temperature of device surface and the ambient air, respectively. Since ϵ and ϵ_amb_ vary significantly from case to case, we considered both the device and ambient as a good radiator, namely, ϵ = ϵ_amb_ = 1, in Fig. 1B. Meanwhile, heat source was ignored (i.e., *P*_heat_ = 0) in Fig. 1B to directly compare the contributions from radiative and nonradiative heat transfer. The ambient temperature was set as *T*_amb_ = 25°C. The nonradiative heat transfer coefficient was chosen as *h* = 5 W/m^2^ per kelvin for typical indoor environment. The radiative cooling power intensity of skin can be defined as

$$P_{\mathrm{net}-\mathrm{skin}}=\epsilon_{\mathrm{skin}}(\sigma T^{4}-\epsilon_{\mathrm{amb}}\sigma T_{\mathrm{amb}}^{4})$$

where ϵ_skin_ = 0.97 is the emissivity of human skin.

### Electric field simulation for USRI

Similar to the numerical simulation for porous radiative cooling materials, we use COMSOL to simulate the optical response of USRI. The position and size of functional fillers were randomly generated by COMSOL LiveLink for MATLAB according to their size distributions and volume fractions. The simulated region was limited to 12 μm by 40 μm due to the component’s limitation for a single COMSOL model. The electric field distribution within the interfaces can be obtained using a periodic model consisting of the polymer matrix and functional fillers. The refractive index for polymer matrix was measured by an ellipsometer (J.A. Woollam RC2). The refractive index for TiO_2_ and SiO_2_ was from (*51*). The refractive index for fluorescent pigment was taken as 1.7 (i.e., the refractive index of SrAl_2_O_4_) since minimal rare earth elements were doped. As shown in fig. S36, all functional fillers were randomly distributed within the matrix. Periodic boundary conditions were applied on the top and bottom sides. Two ports as well as perfect matching layer (PML) boundary conditions were applied for the left and right sides. A plane wave at normal incident is applied as light source.

### Temperature simulation

We used COMSOL to simulate the temperature distribution for resistance wire. The entity of heating wire was imported from its AutoCAD file. A bottom layer PI, a top layer USRI, and two pieces of tape attached to the bottom of PI were then generated. The boundary conditions for all the outer surfaces in the model are heat fluxes including surface radiation based on the emissivity and air convection based on a nonradiative heat transfer coefficient *h*. The model is shown in fig. S10A. As seen in Fig. 3 (D and E), the simulation results matched well with the experiments at the nonradiative heat transfer coefficient of *h* = 15 to 25 W/m^2^ per kelvin. The temperature distribution in fig. S10A can be obtained at *h* = 20 W/m^2^ per kelvin, which matched well with the experiments (Fig. 3F). To reveal the superiority of low thermal conductivity on device stability, a USRI-PCB-Ecoflex three-layer structure was constructed as shown in fig. S7A. Heat flux with an external forced convection at 25 m/s and temperature fixed at skin temperature were adopted as the boundary conditions. The temperature distribution at equilibrium and time-domain temperature evolutions are shown in fig. S7 (B to D). Significant temperature gradient can be observed in USRI to minimize the influence of the external heat source. Lower thermal conductivity can reduce the final temperature of the PCB surface (fig. S7C). Moreover, with the input wind speed as a square wave (fig. S7D), the temperature fluctuation of PCB can be sufficiently reduced at lower thermal conductivity.

### Effective solar reflectance of USRI

Since the fluorescent contribution cannot be distinguished using a commercial UV/VIS/NIR spectrometer, we adopted a calorimetric method to quantitatively evaluate the effective solar reflectance (ESR) of USRI (*62*). Assuming an approximated radiative heat transfer coefficient as $h_{r}\approx 4\epsilon \sigma T_{\mathrm{amb}}^{3}$, the energy balance at equilibrium temperature *T*_eq_ can be expressed as

$$\left(1-\mathrm{ESR})I_{\mathrm{solar}}≅h(T_{\mathrm{eq}}-T_{\mathrm{amb}})+h_{r}(T_{\mathrm{eq}}-T_{\mathrm{atm}}\right)$$

where *I*_solar_ is the solar intensity, *T*_atm_ is the temperatures of the atmosphere, and *T*_amb_ ≈ *T*_atm_. The relationship between the equilibrium temperature and effective solar reflectance (or solar reflectance if no fluorescent contribution) would be

$$T_{\mathrm{eq}}≅\frac{\left[(1-\mathrm{ESR})I_{\mathrm{solar}}+(h+h_{r})T_{\mathrm{amb}}\right]}{h+h_{r}}=a\;\cdot \mathrm{ESR}+b$$

where *a* and *b* are the simplified environmental parameters. Through linear fitting of the equilibrium temperature and solar reflectance of nine reference samples (R1 to R9, no fluorescence pigments; see table S6), we obtained the relationship as *T*_eq_ = − 0.085 ∙ ESR + 31.69 and thereby the fitted ESR of 0.9126 for USRI.

### Statistics

Standard errors in plots are represented by error bars. The Student’s two-tailed, unpaired *t* test was adopted to compare two groups with the corresponding *P* value. Each asterisk in a plot represents a significant difference between two data groups (*P* < 0.05), unless additionally specified in the figure caption.

## Supplementary Materials

### This PDF file includes:

Note S1 Figs. S1 to S36 Tables S1 to S6

## Notes

View/request a protocol for this paper from *Bio-protocol*.
