# Supplementary material for: Ultrathin, soft, radiative cooling interfaces for advanced thermal management in skin electronics
Source: Sci Adv. 2023 Apr 7;9(14):eadg1837. doi: 10.1126/sciadv.adg1837 (PMC10081843; doi:10.1126/sciadv.adg1837)
Supplement: Supplementary file 1 — Note S1 Figs. S1 to S36 Tables S1 to S6 [file sciadv.adg1837_sm.pdf]

Science Advances
AAAS

Supplementary Materials for

Ultrathin, soft, radiative cooling interfaces for advanced thermal management in skin electronics

Jiyu Li *et al.*

Corresponding author: Xinge Yu, xingeyu@cityu.edu.hk; Dangyuan Lei, dangylei@cityu.edu.hk

Sci. Adv. **9**, eadg1837 (2023)
DOI: 10.1126/sciadv.adg1837

This PDF file includes:

Note S1
Figs. S1 to S36
Tables S1 to S6

Note S1: Constituent effects and optimization of USRI

The spectral properties of our USRI are intimately influenced by the volume fractions of functional fillers, which have been optimized both theoretically and experimentally. To reveal the constituent effects and find optimal volume fractions, we adopted a statistical Monte Carlo method incorporating the analytical Mie theory, which has been proved to be robust and effective for typical matrix-filler systems. The spectral responses of our cooling interfaces are obtained by solving the Radiative Transfer Equation with the scattering and absorption efficiencies of the composite, in which the optical constants of all fillers and matrix are required. The refractive indices of TiO_2 and SiO_2 are obtained from Ref. 51. The refractive index of poly-styrene-acrylic within the solar spectral range was measured as shown in fig. S1A, while corresponding data for the infrared band is missing in the literature. Therefore, we took the infrared refractive index of PMMA for poly-styrene-acrylic in our calculations since they exhibit a similar infrared emissivity (fig. S1C).

The calculated overall solar reflectance and infrared emittance of USRI under varied volume fractions of TiO_2 nanoparticles and hollow SiO_2 microspheres are shown in fig. S2. Both parameters are larger at higher volume fractions of TiO_2 nanoparticles ($f_{V\mathrm{TiO2}}$) and approach their maxima at $f_{V\mathrm{TiO2}} > 10\%$. Surprisingly, increasing the volume fraction of hollow SiO_2 microspheres ($f_{V\mathrm{SiO2}}$) can only slightly enhance the infrared emittance when $f_{V\mathrm{TiO2}} < 5\%$ (fig. S2B), but barely contribute to the overall solar reflectance (fig. S2A). Therefore, we believe that the hollow SiO_2 microspheres can only assist the overall infrared emission of the cooling interface and dominate its thermal conductivity reduction. Our experimental results reveal the same trend of spectral properties under varied $f_{V\mathrm{TiO2}}$ and $f_{V\mathrm{SiO2}}$ as shown in fig. 2C&D. The small drop in the measured solar reflectance at $f_{V\mathrm{TiO2}} > 10\%$ may be ascribed to the increased UV absorption of TiO_2 nanoparticles. In addition, In addition, the measured infrared emittance is ~0.02 higher than the calculation results, which could be due to the difference in the refractive indices of PMMA and poly-styrene-acrylic. A slightly higher solar reflectance can be observed at $f_{V\mathrm{SiO2}} \sim 40\%$. According to these results, a $f_{V\mathrm{TiO2}}$ of ~ 10% and a $f_{V\mathrm{SiO2}}$ of ~ 25% were selected in our USRI.

The fluorescence contribution needs to be characterized by effective solar reflectance (*ESR*) since it cannot be analyzed by either a commercial UV/Vis/NIR spectrometer or the Monte Carlo method. Therefore, only experimental optimization was executed and the results are shown in fig. S2E&F. It can be seen that the solar reflectance is larger at moderate volume fractions of fluorescent pigments (i.e., $6\% < f_{V\mathrm{fluo}} < 10\%$) while the infrared emittance remains almost unchanged as $f_{V\mathrm{fluo}}$ varies. Note that the solar reflectance results shown in fig. S2E were measured after keeping the five samples in a dark box for 8 hours in order to release their photoluminescence completely. Furthermore, we measured the temperatures of the five samples under direct sunlight exposure. As shown in fig. S2F, increasing $f_{V\mathrm{fluo}}$ results in lower

temperatures, corresponding to higher *ESR*. Therefore, a $f_{V\text{fluo}}$ of ~9% was used in our USRI.

In addition to spectral properties, the mechanical performance of our USRI were also improved. Different from our previously reported SSRC (Ref. 56), which used the same matrix and fillers as USRI for building applications, we applied less hollow SiO_2 microspheres to reduce the total volume fraction and added more film forming agent to improve the flexibility of USRI. The strain-stress results of USRI and SSRC are illustrated in fig. S4A. The Young's modulus, stretchability and toughness of USRI were measured as 21.2 MPa, 110% and 1.414 MJ/m^{-3}, respectively.

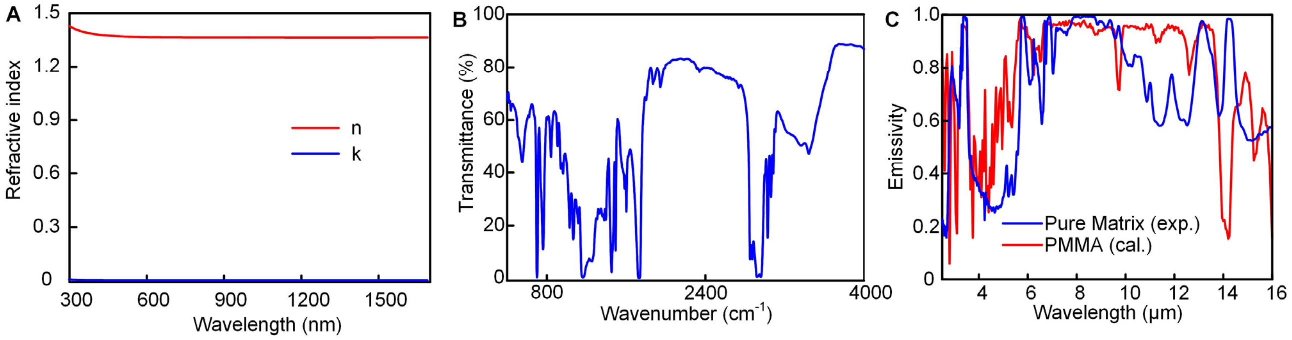

Supplementary Figure 1. Optical properties of polymer matrix. (A) Measured refractive index of polymer matrix. (**B**) FTIR transmittance spectrum of 30 μm-thick polymer matrix. (**C**) Comparison between the measured emissivity of poly-styrene-acrylic (Pure matrix) and calculated emissivity of PMMA with the same thickness of 200 μm.

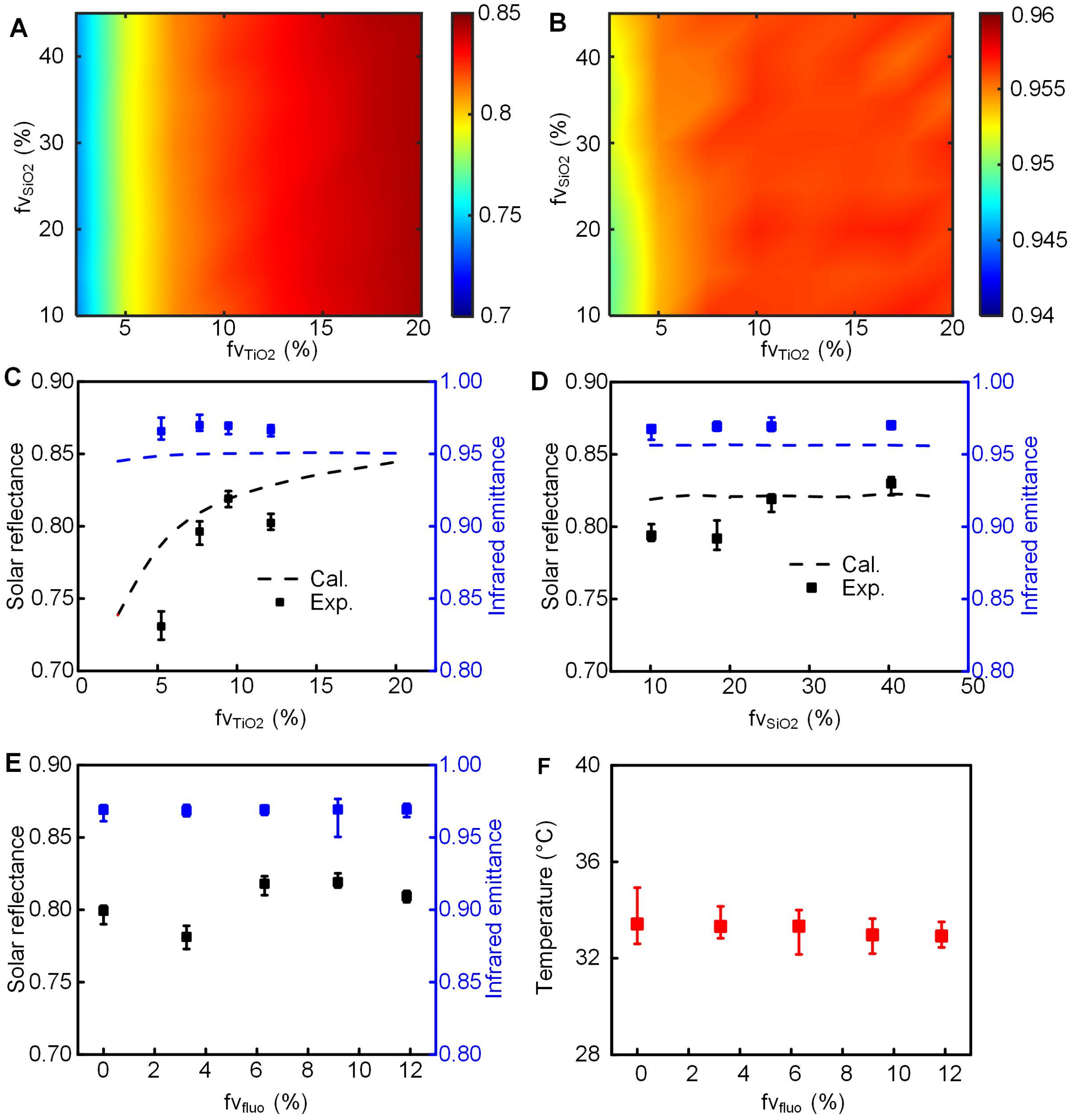

Supplementary Figure 2. The effect of constituent fillers. (**A**) overall solar reflectance and (**B**) infrared emittance of cooling interface with varying volume fractions of TiO_2 nanoparticles and hollow SiO_2 microspheres. (**C**) Experimental verification of the constituent effect of TiO_2 nanoparticles. The volume fraction of hollow SiO_2 microspheres is fixed at 25%. (**D**) Experimental verification of the constituent effect of hollow SiO_2 microspheres. The volume fraction of TiO_2 nanoparticles is fixed at 10%. € Measured solar reflectance and infrared emittance with different volume fractions of fluorescent pigments. (**F**) Measured temperature of all samples €(**E**) under direct sun exposure.

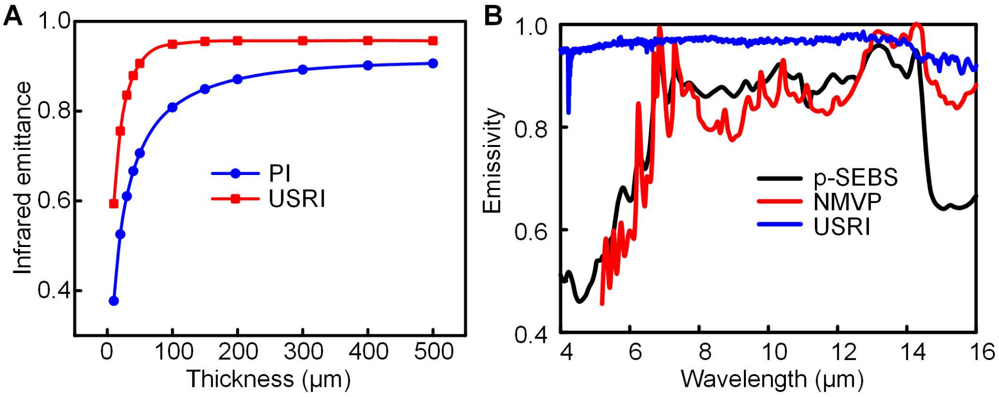

Supplementary Figure 3. Superiority of USRI on infrared emissivity. (**A**) Calculated infrared emittance of PI and USRI with varying thickness. (**B**) Infrared emissivity of previously reported radiative cooling materials (p-SEBS for Ref. 45 and NMVP for Ref. 17) for wearable electronics and our USRI.

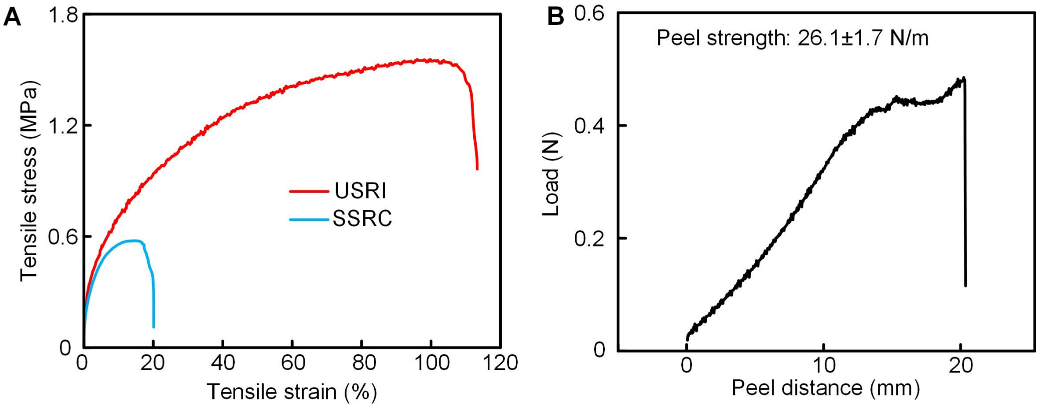

Supplementary Figure 4. Stretchability and peeling force measurement of USRI. (**A**) Stain-stress measurement of USRI and SSRC. (**B**) The peeling force variation of USRI-integrated conductive interconnects measured by 90 degree peeling test.

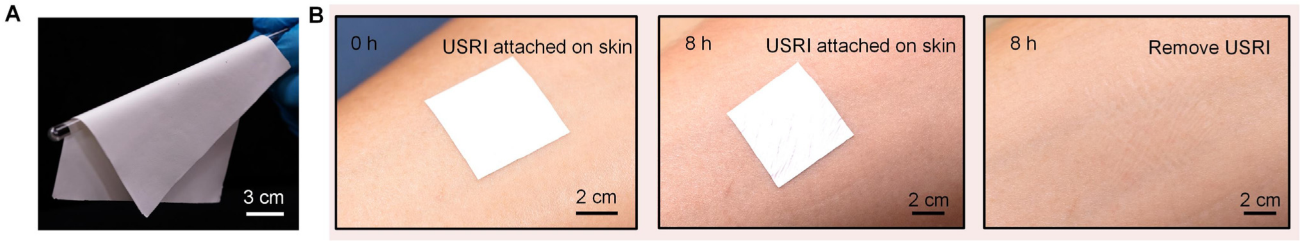

Supplementary Figure 5. Biocompatibility testing of USRI. (**A**) Image of the USRI hanging on a glass rod. (**B**) Picture of a volunteer before and after wearing a USRI on forearm for 8 hours.

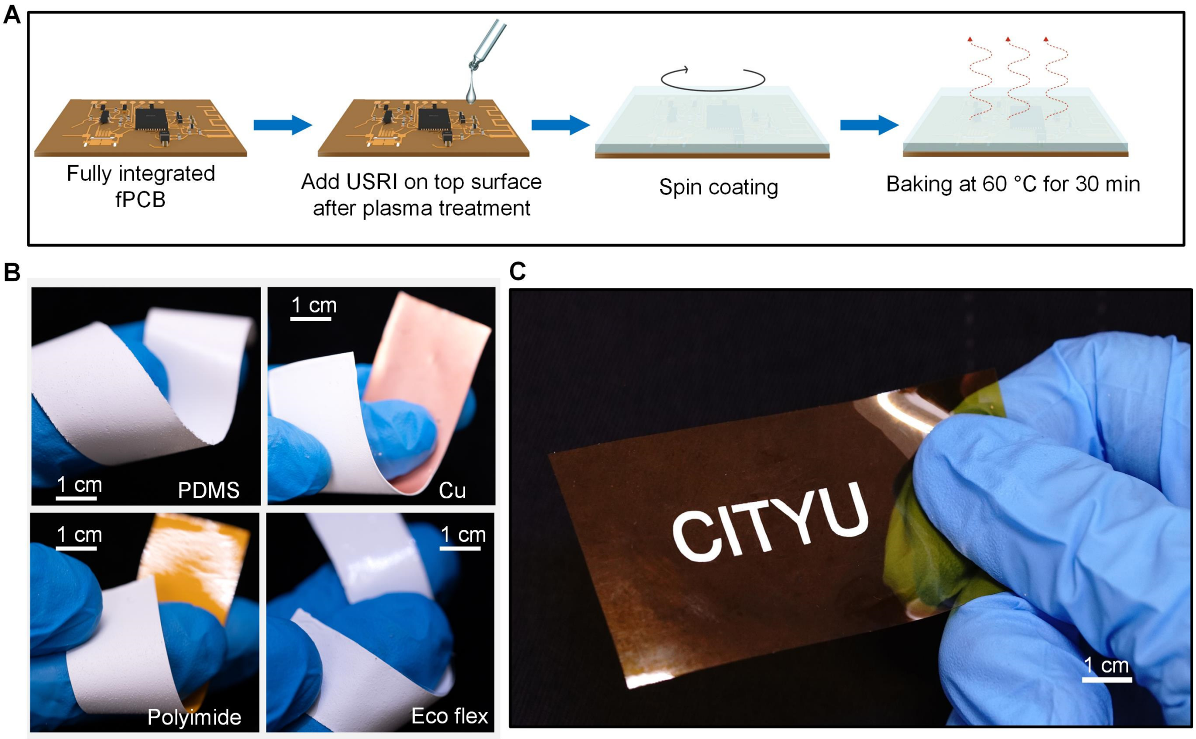

Supplementary Figure 6. Integration of USRI on series of typical substrate/ circuit materials. (A) Integration process of USRI on wearable and flexible electronics as an encapsulation layer based on spin coating. (**B**) Picture of USRI adhering on a series of materials including, PDMS, copper, polyimide and Eco flex under bending. (**C**) Photographs of the "CITYU" pattern of mask-sprayed USRI on PI substrate.

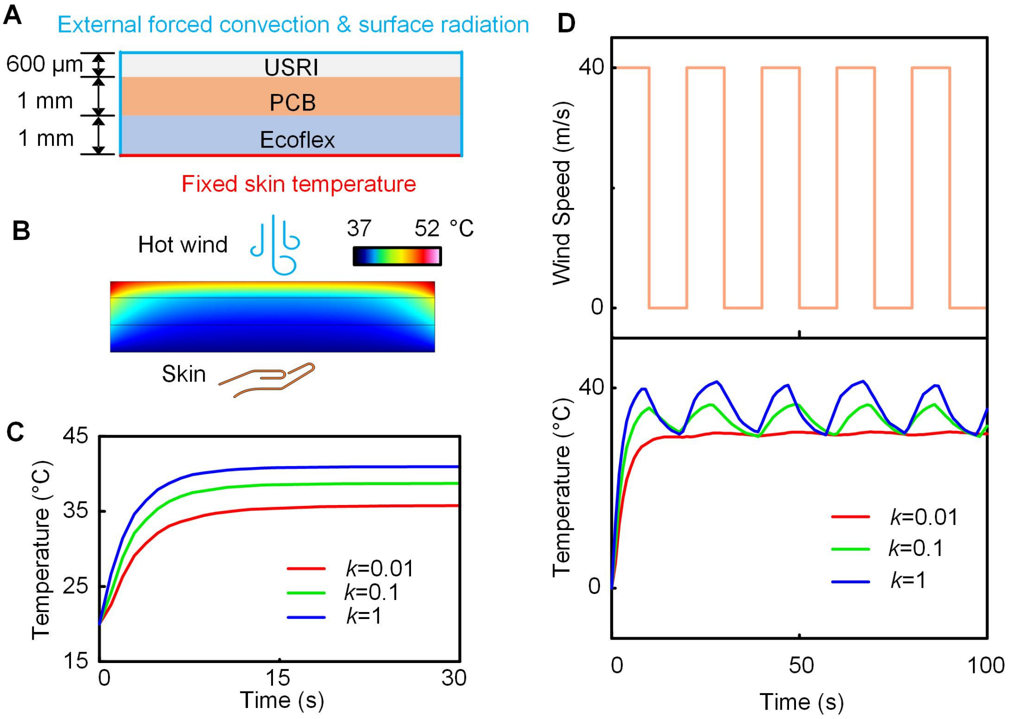

Supplementary Figure 7. Temporal temperature simulation for USRI integrated PCB. (A) Simulation model for time-domain temperature distribution . (**B**) Simulated cross-sectional temperature distribution of USRI-PCB-Ecoflex structure under hot wind. **(C)** Time-domain temperature evolution for the temperature of PCB middle layer under

continuous hot wind. **(D)** Hot wind in the form of square wave (top) and corresponding time-domain temperature evolution for the temperature of PCB middle layer (bottom). The thermal conductivity of USRI in **(C)** and **(D)** varies from 0.01 to 1 W/(m·K).

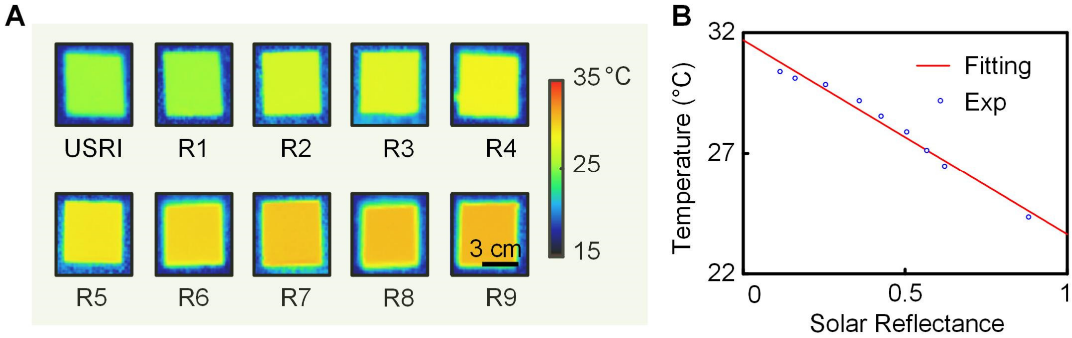

Supplementary Figure 8. ESR fitting for USRI. (A) IR image of USRI and 9 reference interfaces under outdoor sunlight exposure after 30 minutes. **(B)** Fitted relationship between solar reflectance and coating temperature from 22 to 32 °C. The effective solar reflectance of USRI is fitted as 0.9126.

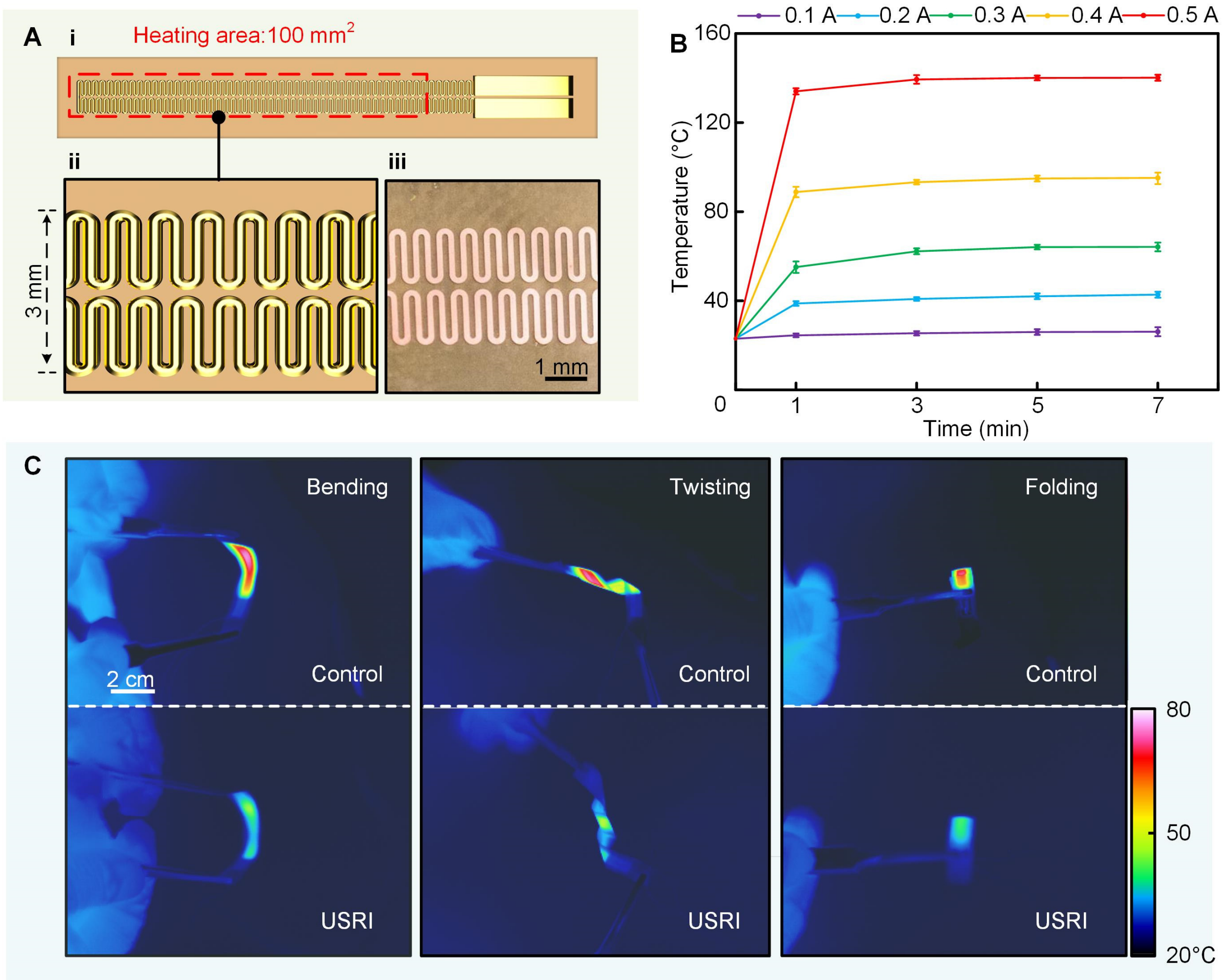

Supplementary Figure 9. Appling USRI on flexible heating wire. (A) Heating area of flexible heating wire(i), enlarge view of heating wire (ii) and its optical image (iii). **(B)** Temperature variation of flexible heating wire working under a series of input currents for 7 minutes. The thermal equilibrium temperature of flexible heating wire was achieved after 3

minutes. **(C)** IR image of flexible heating wire with and without USRI working under bending, twisting and folding (input current: 0.3 A).

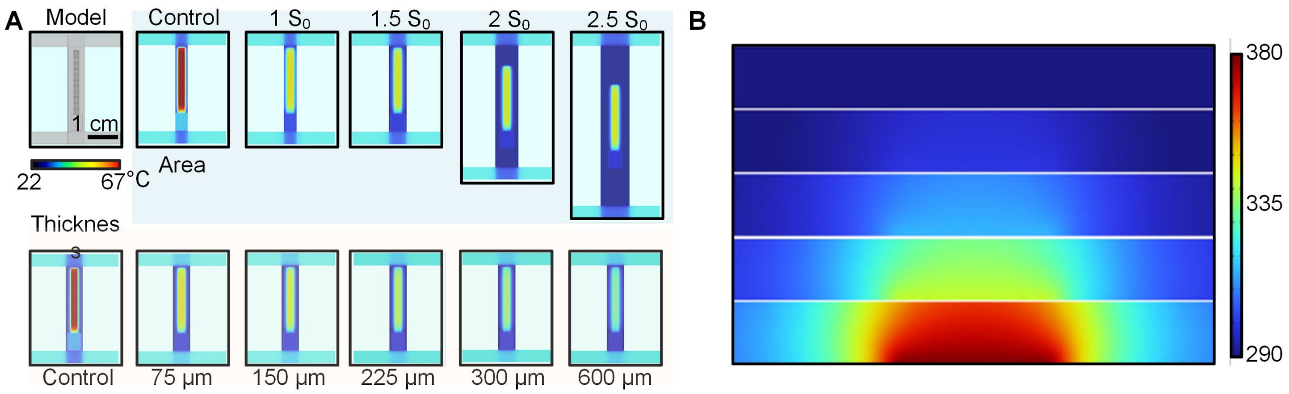

Supplementary Figure 10. Temperature simulation of USRI- integrated flexible heating wire with different covering thickness and area. (A) COMSOL model and simulated results of temperature distribution in Figure 2F. **(B)** Cross-sectional temperature distribution of flexible heating wire coated with 600-µm-thick USRI. The input currents vary from 0.1 (top) to 0.5 A (bottom).

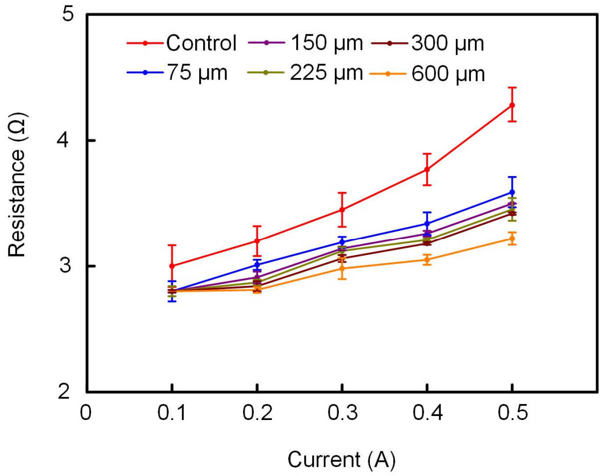

Supplementary Figure 11. Resistance variation of flexible heating wire along with the increasing of input current.

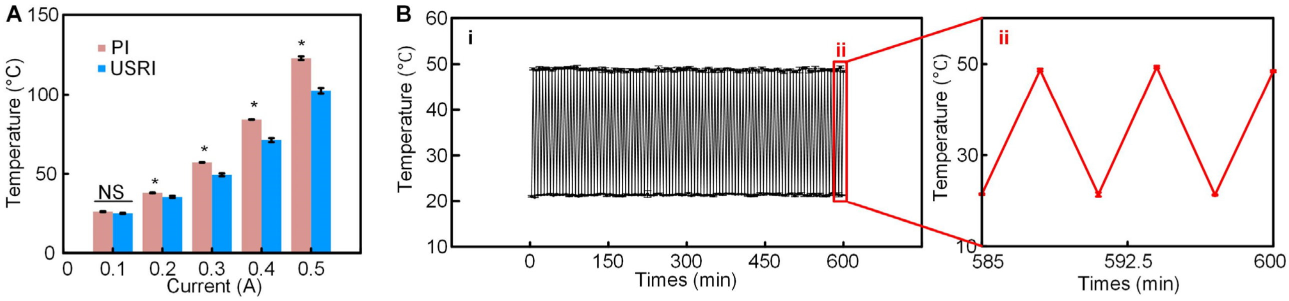

Supplementary Figure 12. The investigation of the cooling performance of USRI compared with polyimide and thermal stability test. (A) Temperature of the flexible heating wire coated with PI and USRI. (B) Temperature of USRI-integrated flexible heating wire under 100 times of heating/cooling cycles. The thickness and area of USRI and the input current are H=150 µm, S=1.5S_0 and 0.3A, respectively.

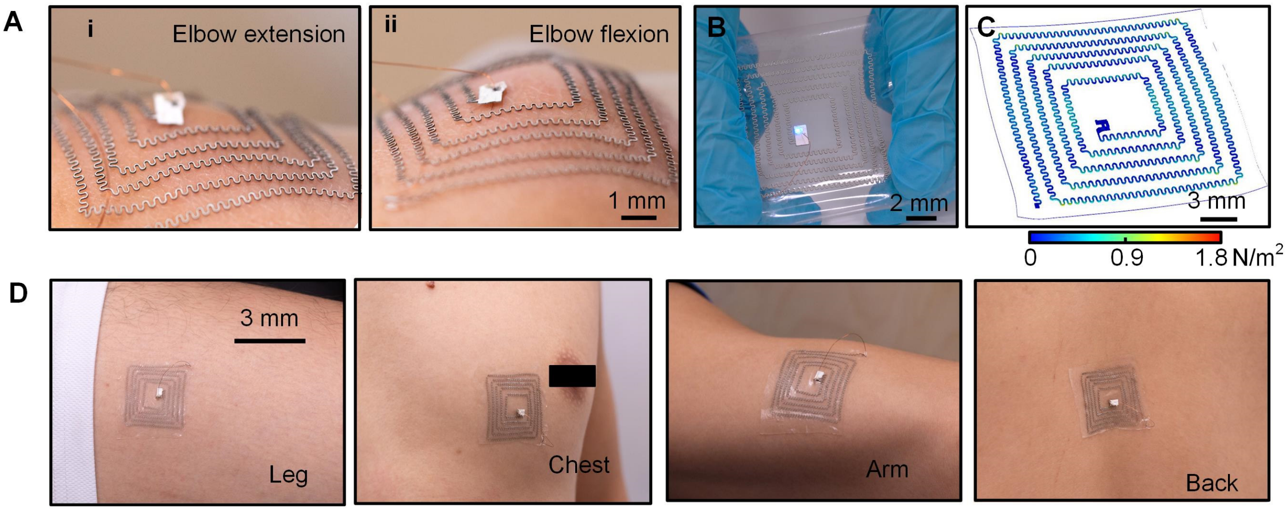

Supplementary Figure 13. Appling the wireless and stretchable LED lighting system on human skin. (**A**) Photograph of LED lighting system adhering on elbow under extension (i) and flection (ii). **(B)** Photograph of the LED lighting system integrated with USRI under stretching. **(C)** Mechanical simulation of lighting system with USRI under stretching with tensile stress of 5N/m^2. The color represents the equivalent strain. (**D**) Photograph showing that the LED lighting system integrated with USRI can adhesive seamless on leg, chest, arm and back.

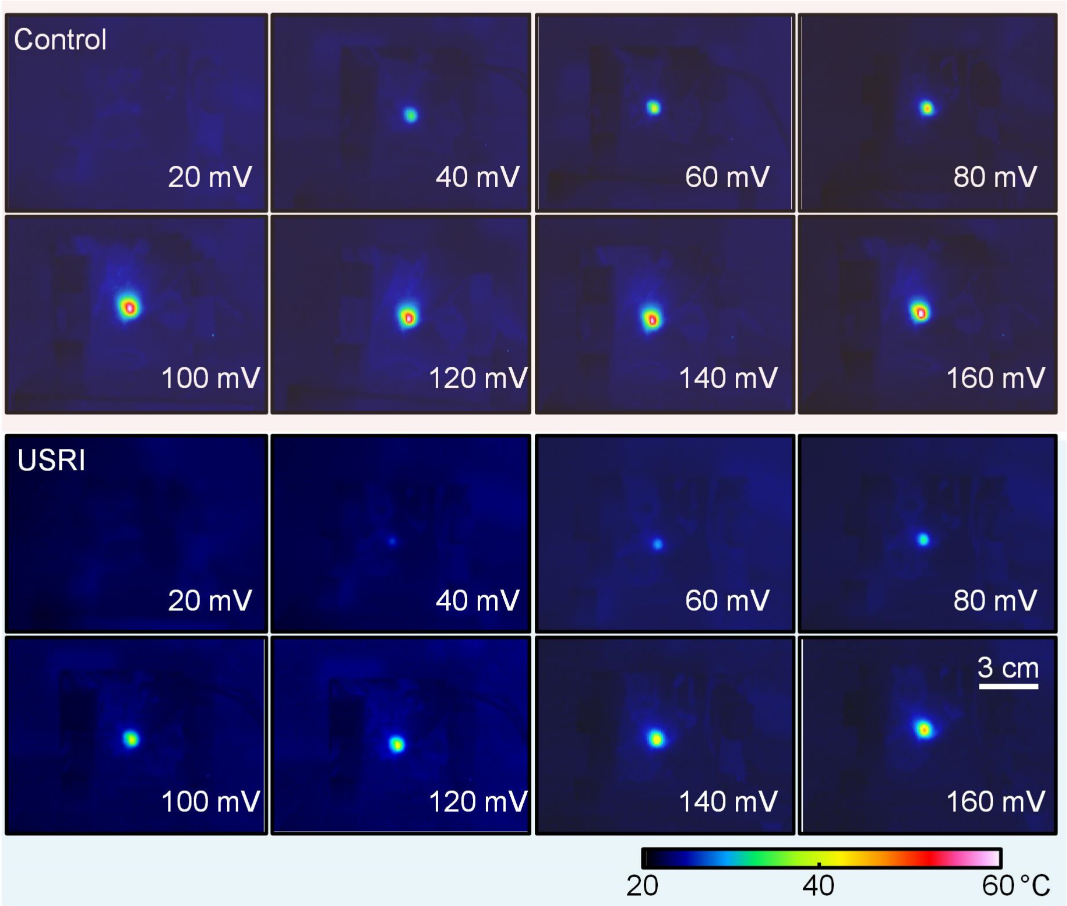

Supplementary Figure 14. IR image of wireless and stretchable LED lighting system with and without USRI working within the input voltage range from 20 mV to 160 mV.

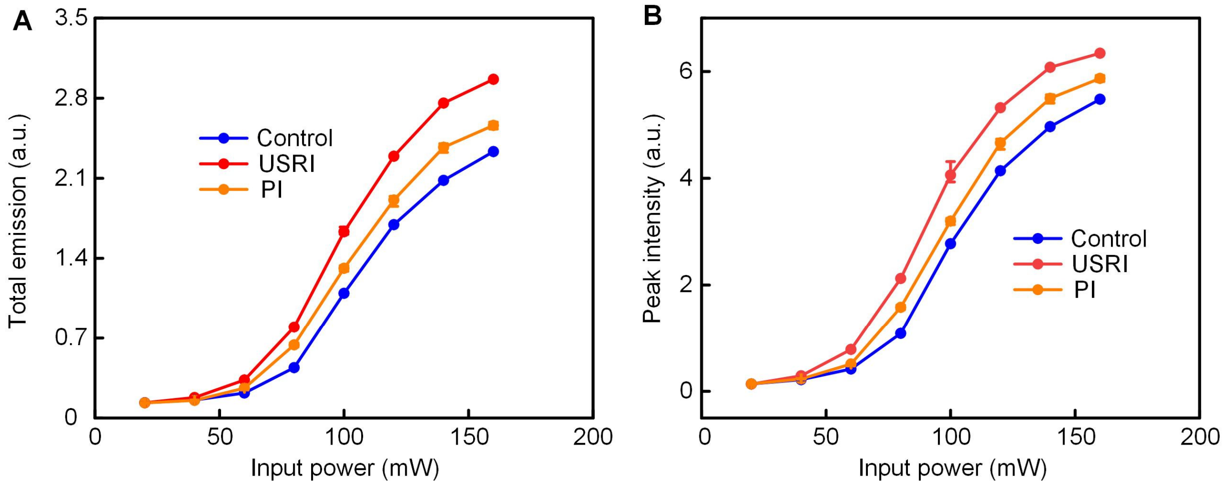

Supplementary Figure 15. Emission investigation of the LED. Total emission (**A**) and peak intensity (**B**) of LED light from bare device (control) and devices encapsulated by USRI and PI.

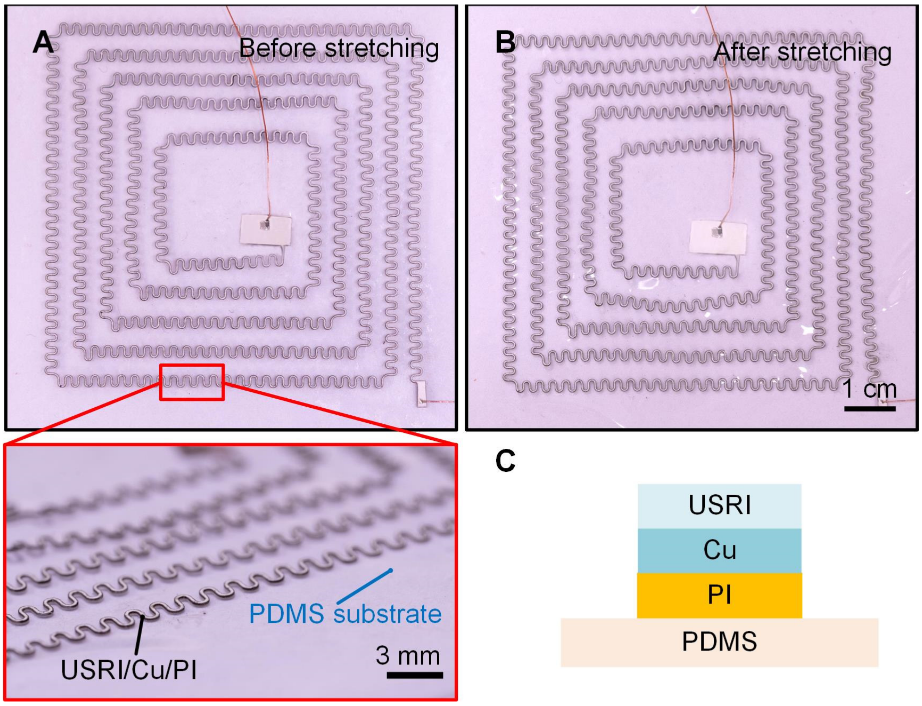

Supplementary Figure 16. Stretchability test of RF wireless LED lighting system. Photograph of the RF wireless-based device integrated with USRI before (**A**) and after (**B**) stretching. (**C**) Schematic figure for the cross section of the device.

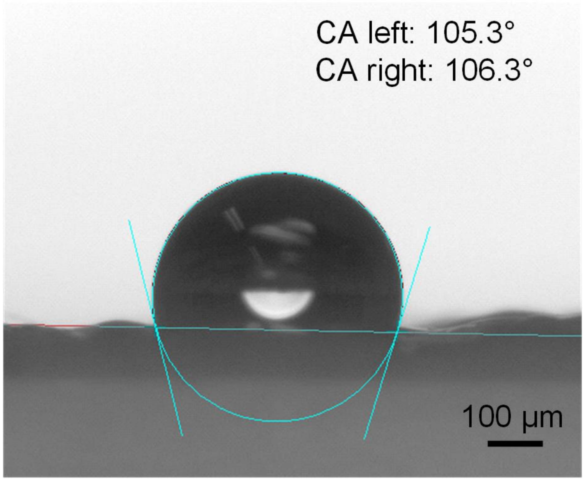

Supplementary Figure 17. Contact angle measurement of USRI after hydrophobic treatment.

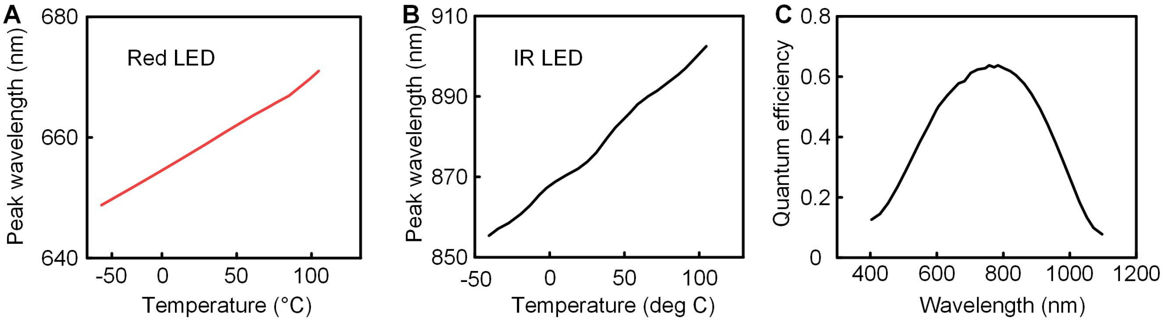

Supplementary Figure 18. Properties of light sources and sensor for PPG sensing platform. (A, B) The relationship between the peak wavelengths of red LED **(A)**, IR LED **(B)** and temperature. **(C)** The relationship between the quanta efficiency and wavelength for photodiode sensor.

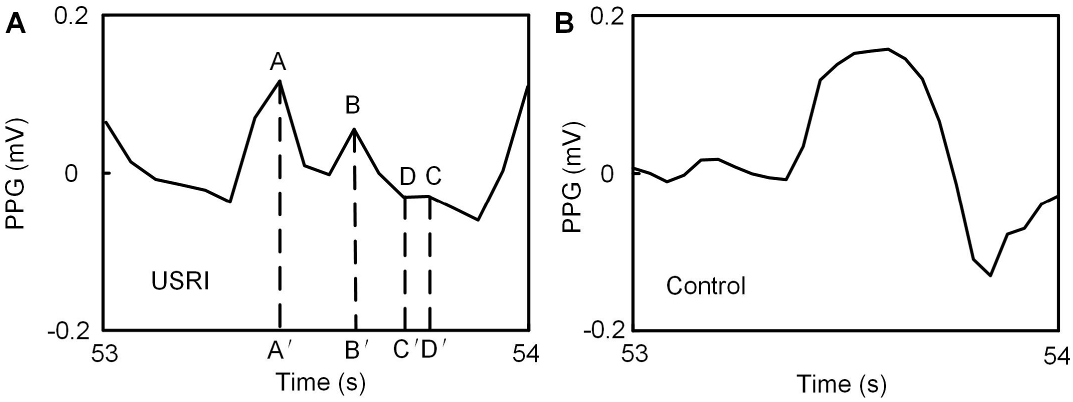

Supplementary Figure 19. Further magnified PPG signals. Signal collected from USRI group (**A**) and control group (**B**) after sunlight exposure (53s to 54s).

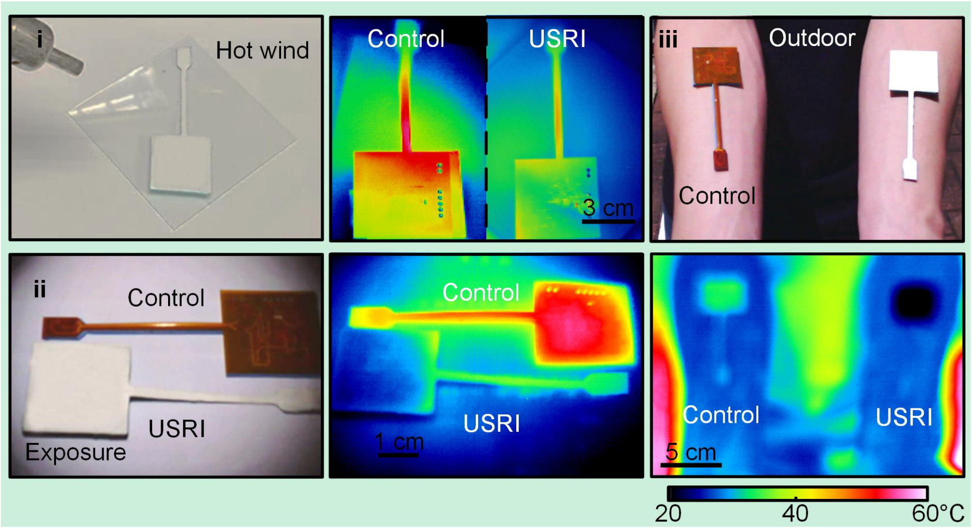

Supplementary Figure 20. Thermal stability test of USRI-integrated PPG sensing system in various of environment. IR camera measurements of the temperature of back side of PPG sensing system under hot wind (i), sunlight exposure (1500 W/m^2) (ii) and outdoor scene (iii).

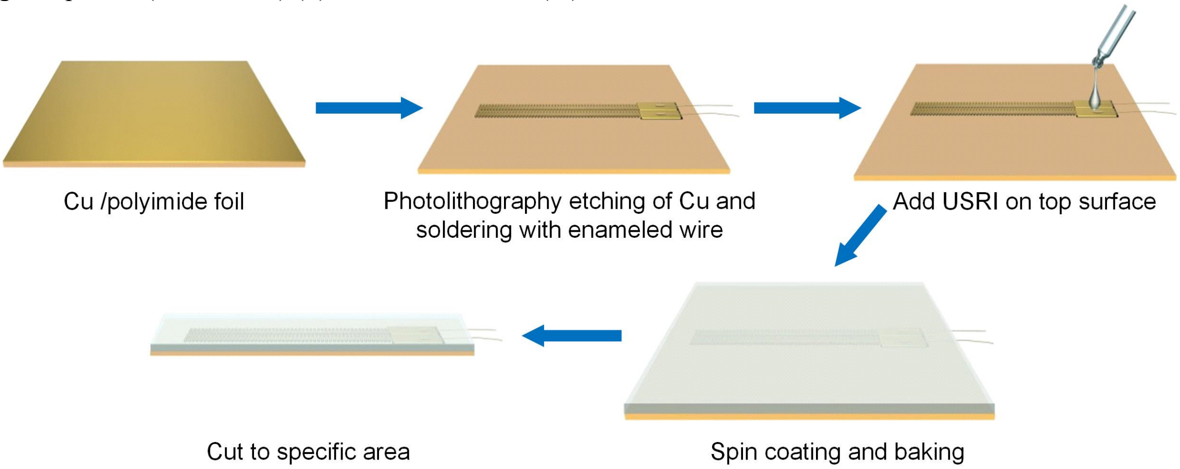

Supplementary Figure 21. Fabrication process of flexible heating wire integrated with USRI.

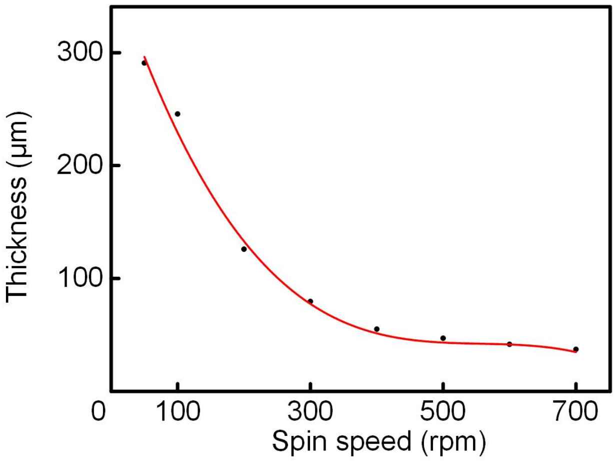

Supplementary Figure 22. USRI spin speed vs thickness (on polyimide layer, speed 0 to 700 rpm).

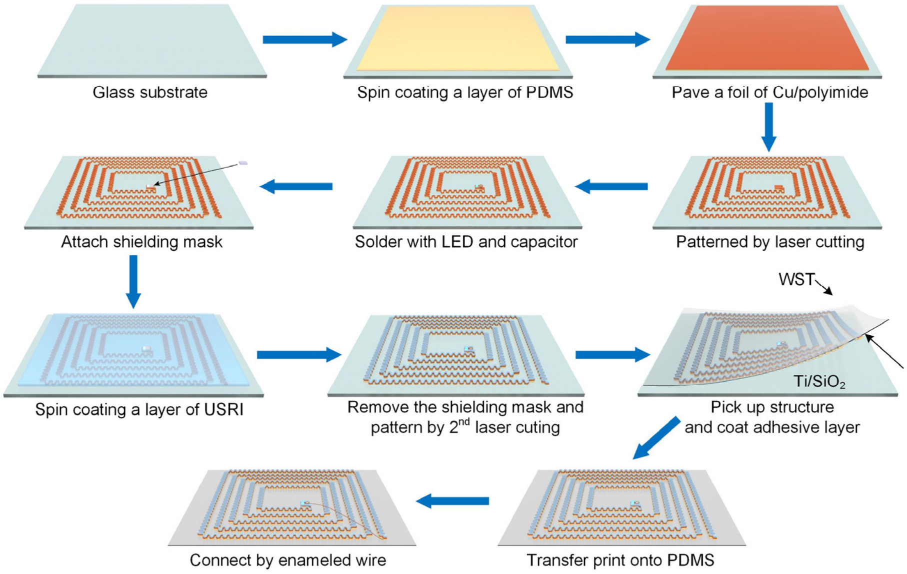

Supplementary Figure 23. Fabrication process of RF wireless-based lighting system integrated with USRI.

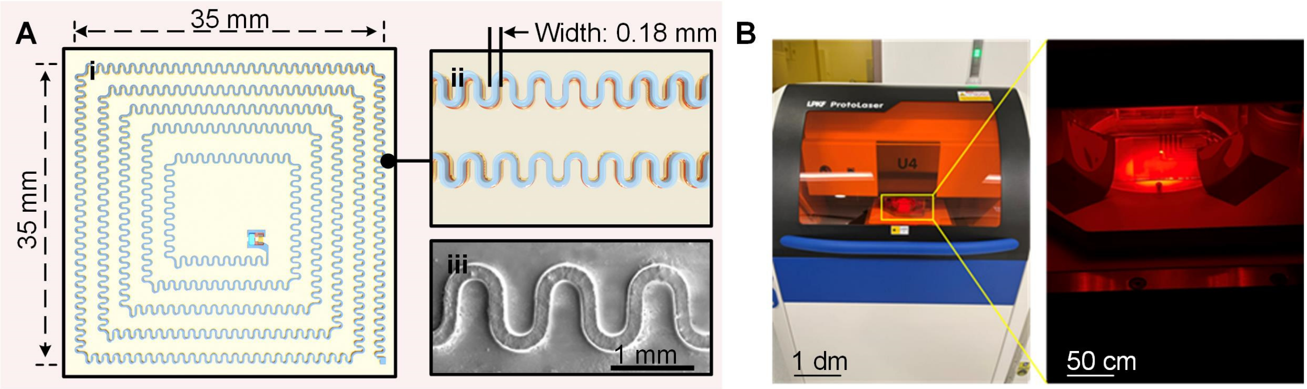

Supplementary Figure 24. Design layout of lighting system and experiment setup for laser cutting (**A**)Dimensions of lighting system including side length (i), coil width (ii) and SEM image of single serpentine coil wire (iii). (**B**) Experiment setup for laser cutting and enlarge view of fabrication of serpentine coil.

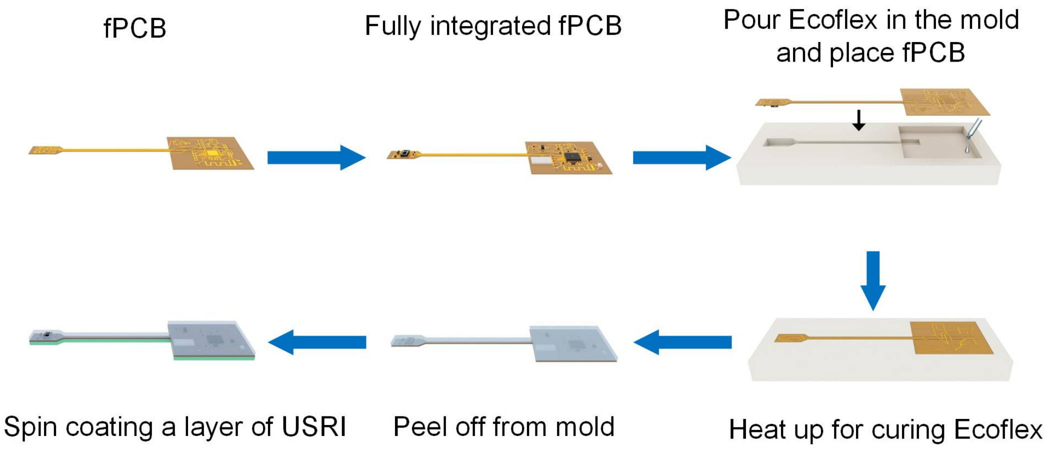

Supplementary Figure 25. Fabrication process of wireless PPG sensing system integrated with USRI.

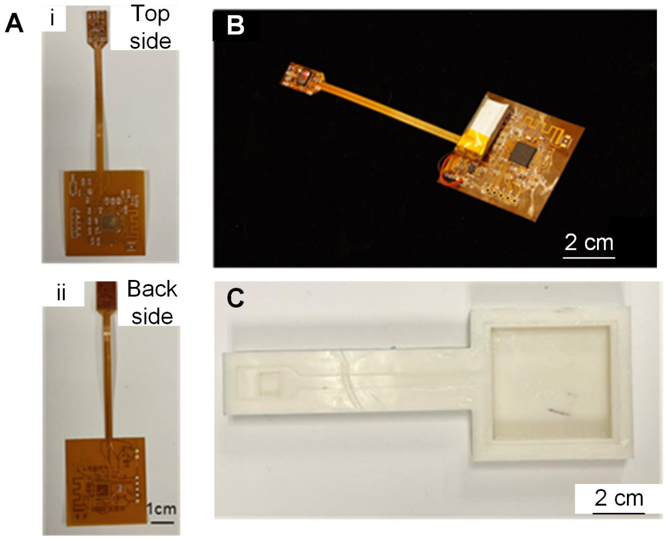

Supplementary Figure 26. Photographs of the wearable PPG sensor. Top side (i) and back side (ii) of fPCB **(A)** and the finger like PPG sensor working without any encapsulation **(B)** and image of mold **(C)**.

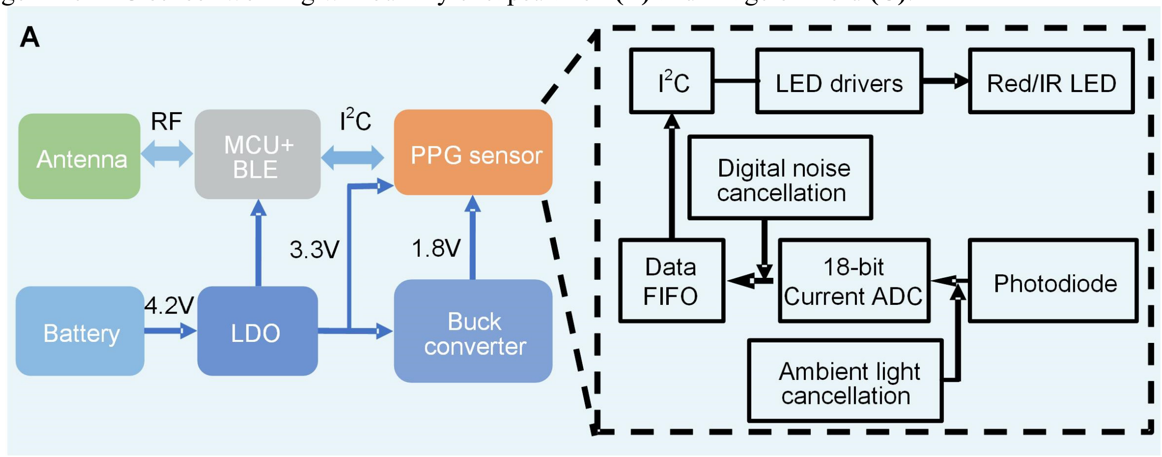

Supplementary Figure 27. Circuit diagram for finger like wireless PPF signal sensor system.

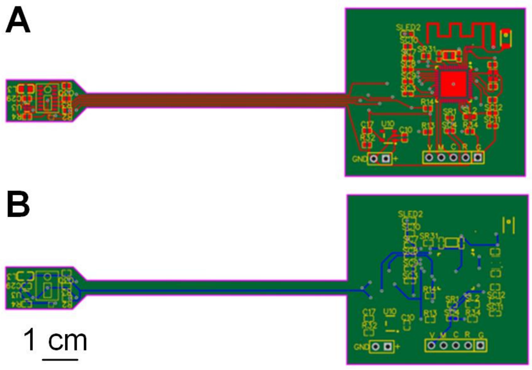

Supplementary Figure 28. PCB design of the finger-like wireless PPG sensing system. (A) Top layer of copper. **(B)** Bottom layer of copper.

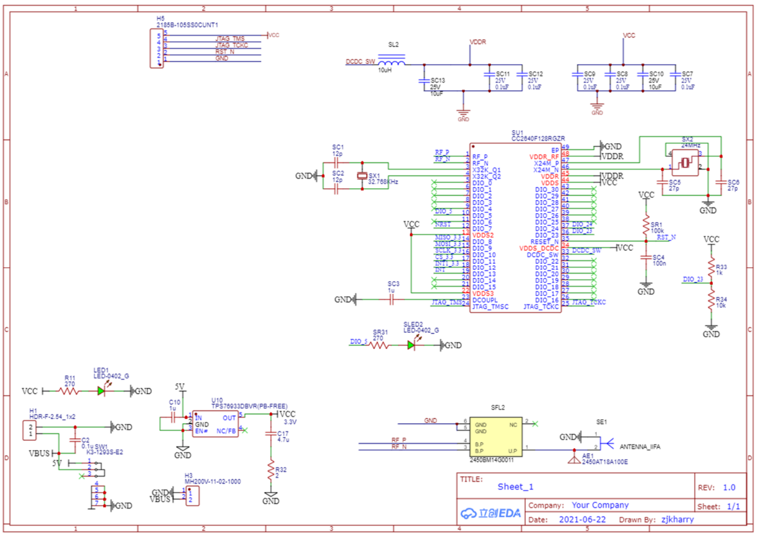

Supplementary Figure 29. Schematic design of the wireless PPG sensing system (main).

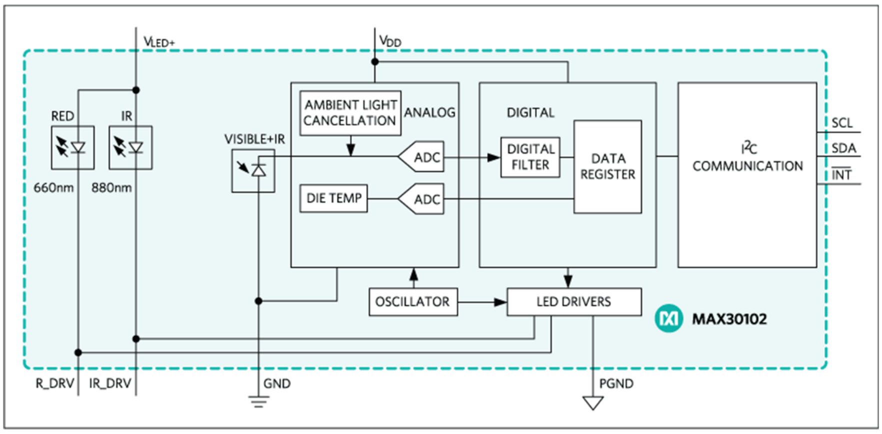

Supplementary Figure 30. Circuit diagram for PPG sensor (MAX30102).

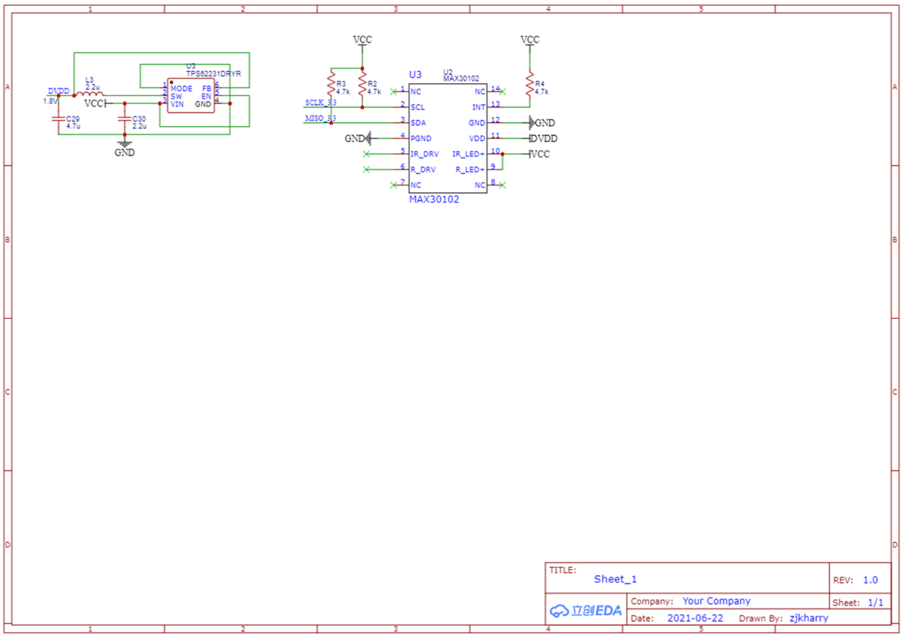

Supplementary Figure 31. Schematic design of the wireless PPG sensing system (PPG sensor).

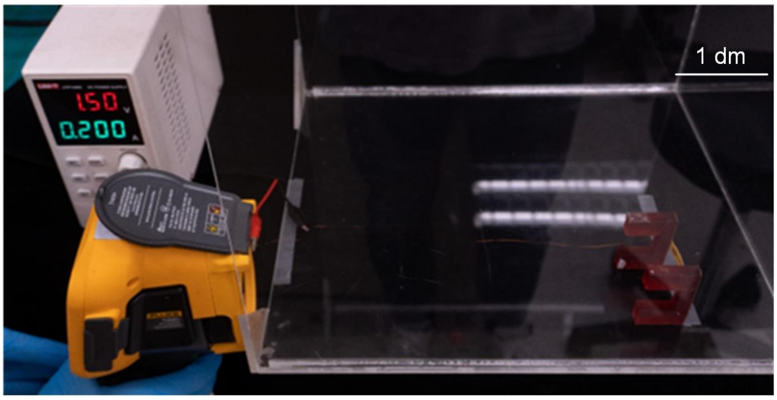

Supplementary Figure 32. Experiment setup for IR camera measurement of temperature variation of flexible heating wire

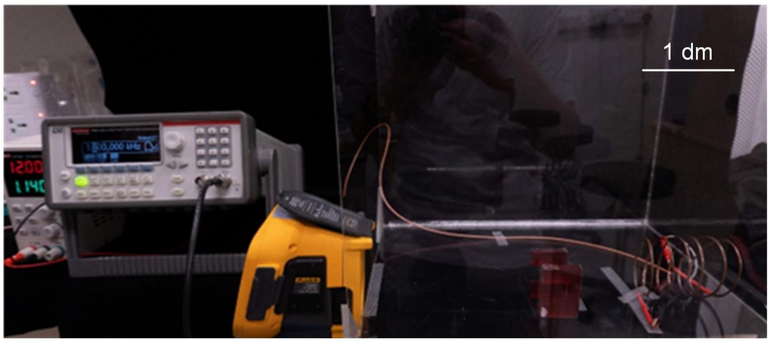

Supplementary Figure 33. Experiment setup for IR camera measurement of wireless and stretchable epidermal LED lighting system

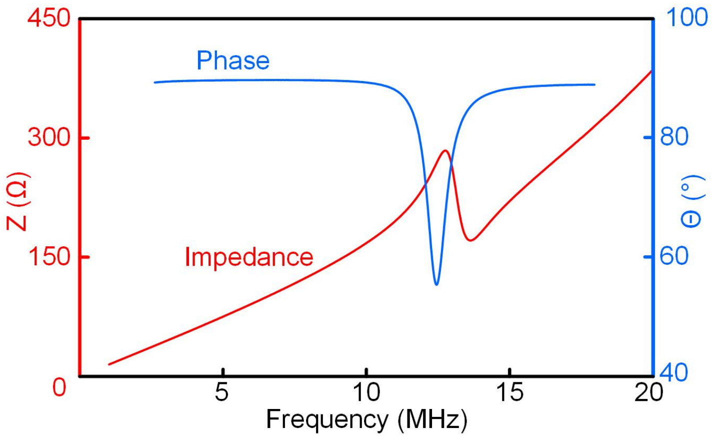

Supplementary Figure 34. Impedance test results of the fabricated wireless and stretchable LED lighting system.

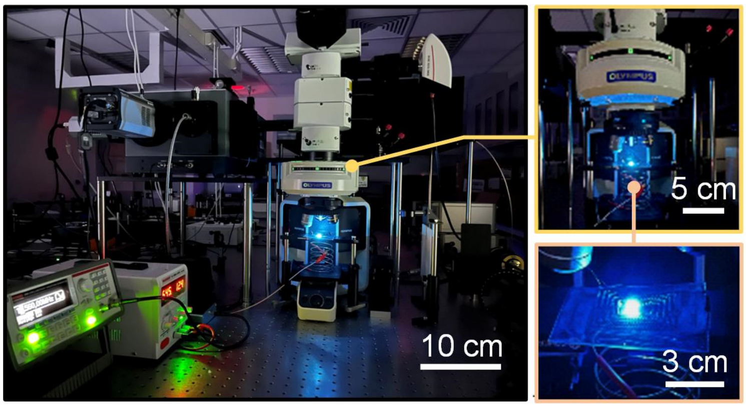

Supplementary Figure 35. Optical image of experimental setup for spectral measurement of fluorescence emitted from lighting system.

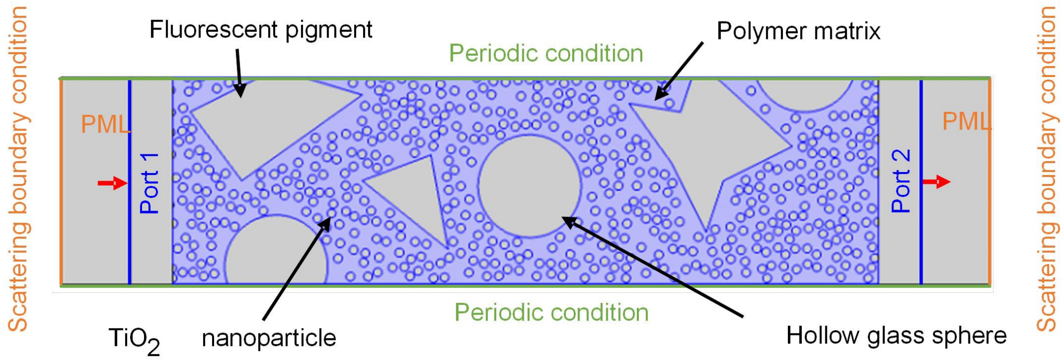

Supplementary Figure 36. Simulation model for electric field distribution in Fig. 2e.

Supplementary table 1. Thermal conductivities of USRI and pure polymer matrix.

	**Thermal conductivity**	
	Measured at (K)	Result (W/(m·K))

USRI	298.73	0.1106
	298.69	0.1115
	298.69	0.1119
	Average	0.1113
Polymer matrix (dried)	298.40	0.2827
	298.43	0.2814
	298.41	0.2832
	Average	0.2824

Supplementary table 2. Overall solar reflectance and infrared emissivity in Fig.2f-g.

	**Polymer Matrix**	**100 µm USRI**	**150 µm USRI**	**200 µm USRI**	**3500 µm USRI**
Solar Reflectance	0.0913	0.7035	0.7663	0.8103	0.8810
Infrared Emissivity	0.7836	0.9529	0.9654	0.9694	0.9708

Supplementary table 3. The peeling strength of USRI integrated with series of typical substrate/circuits materials measured by 90 degree peeling test.

	**PI**	**Cu**	**Ecoflex**	**PDMS**
Peeling strength (N/m)	23.6 ± 1.3	55.4± 4.7	31.3± 2.5	19.2± 1.8

Supplementary table 4. Heart rate data in USRI group and control group collected by PPG sensor under different situations.

Group	**USRI (bpm)**	**Control (bpm)**
Hot wind	61	55
Sun light	53	46
Walking from indoor to outdoor	67	56

Supplementary table 5. Volume fractions of main components for USRI. The additives are ignored since they are minor components.

**Component**	**Density (g/cm^3)**	**Weight (g)**	**Volume fraction (%)**
Polymer matrix (dried)	0.85	45	55.66
TiO_2 nanoparticles	4.23	40	9.94
Fluorescent pigment	3.44	30	9.17
Hollow glass spheres	0.25	6	25.23

Supplementary table 6. Solar reflectance of reference samples for effective solar reflectance fitting.

Sample	UV	vis		NIR	Total
R1	0.0808	0.9383		0.8858	0.8807
R2	0.0855	0.6633		0.6218	0.6215
R3	0.0921	0.6115		0.5589	0.5664
R4	0.0881	0.5395		0.5023	0.5045
R5	0.0861	0.4520		0.4255	0.4254
R6	0.0796	0.3795		0.3593	0.3584
R7	0.0818	0.2499		0.2713	0.2541
R8	0.0819	0.1296		0.1956	0.1601
R9	0.0790	0.0809		0.1479	0.1136
